# Distinct inflammatory profiles distinguish COVID-19 from influenza with limited contributions from cytokine storm

**DOI:** 10.1126/sciadv.abe3024

**Published:** 2020-12-09

**Authors:** Philip A. Mudd, Jeremy Chase Crawford, Jackson S. Turner, Aisha Souquette, Daniel Reynolds, Diane Bender, James P. Bosanquet, Nitin J. Anand, David A. Striker, R. Scott Martin, Adrianus C. M. Boon, Stacey L. House, Kenneth E. Remy, Richard S. Hotchkiss, Rachel M. Presti, Jane A. O’Halloran, William G. Powderly, Paul G. Thomas, Ali H. Ellebedy

**Affiliations:** 1 Department of Emergency Medicine, Washington University School of Medicine, Saint Louis, MO, USA.; 2 Department of Immunology, St. Jude Children’s Research Hospital, Memphis, TN, USA.; 3 Department of Pathology and Immunology, Washington University School of Medicine, Saint Louis, MO, USA.; 4 Department of Internal Medicine, Washington University School of Medicine, Saint Louis, MO, USA.; 5 Bursky Center for Human Immunology and Immunotherapy Program, Washington University School of Medicine, Saint Louis, MO, USA.; 6 Department of Critical Care, Missouri Baptist Medical Center, Saint Louis, MO, USA.; 7 Department of Pediatrics, Washington University School of Medicine, Saint Louis, MO, USA.; 8 Department of Anesthesiology, Washington University School of Medicine, Saint Louis, MO, USA.; 9 Department of Surgery, Washington University School of Medicine, Saint Louis, MO, USA.

## Abstract

We pursued a study of immune responses in coronavirus disease 2019 (COVID-19) and influenza patients. Compared to patients with influenza, patients with COVID-19 exhibited largely equivalent lymphocyte counts, fewer monocytes, and lower surface human leukocyte antigen (HLA)–class II expression on selected monocyte populations. Furthermore, decreased HLA-DR on intermediate monocytes predicted severe COVID-19 disease. In contrast to prevailing assumptions, very few (7 of 168) patients with COVID-19 exhibited cytokine profiles indicative of cytokine storm syndrome. After controlling for multiple factors including age and sample time point, patients with COVID-19 exhibited lower cytokine levels than patients with influenza. Up-regulation of IL-6, G-CSF, IL-1RA, and MCP1 predicted death in patients with COVID-19 but were not statistically higher than patients with influenza. Single-cell transcriptional profiling revealed profound suppression of interferon signaling among patients with COVID-19. When considered across the spectrum of peripheral immune profiles, patients with COVID-19 are less inflamed than patients with influenza.

## Teaser

Immunologic evaluation shows that most COVID-19 patients exhibit targeted immunosuppression compared to patients with influenza.

## INTRODUCTION

Infection with severe acute respiratory syndrome coronavirus 2 (SARS-CoV-2) causes coronavirus disease 2019 (COVID-19). Acute respiratory failure occurs in a subset of patients with COVID-19 (*1*–*3*). Respiratory failure has occurred in as many as 8% of individuals testing positive for infection in the Lombardy region of Italy (*4*). Understanding the etiology of respiratory failure in patients with COVID-19 is critical for determining the best management strategies and pharmacologic targets for treatment. Current management of acute respiratory failure in COVID-19 consists of optimized supportive care (*5*, *6*), primarily through oxygen administration and consideration of endotracheal intubation and mechanical ventilation in the appropriate context (*7*). Recent evidence published in the RECOVERY (Randomized Evaluation of COVID-19 Therapy) trial (*8*) suggests that the administration of high dose steroids in a small subgroup of critically ill patients may reduce mortality.

Cytokine storm syndrome (CSS) has been proposed as underlying the etiology of respiratory failure in patients with COVID-19 (*9*). This model suggests that respiratory failure is related to significant proinflammatory cytokine expression that leads to inflammatory cell recruitment and tissue damage in the lung. Most of the data supporting this hypothesis in COVID-19 come from an early paper that observed high levels of the cytokines interleukin-2 (IL-2), IL-7, IL-10, granulocyte colony-stimulating factor (G-CSF), Interferon γ-induced protein 10 (IP-10), monocyte chemoattractant protein 1 (MCP1), macrophage inflammatory protein–1α (MIP-1α), and tumor necrosis factor α (TNFα) in a small cohort of patients with COVID-19 being cared for in the intensive care unit (ICU). The level of these cytokines was increased in the ICU patients compared with a group of patients with COVID-19 that did not require care in the ICU (*3*). The CSS hypothesis for respiratory failure in COVID-19 has recently been challenged, as others have more closely examined data from early studies of cytokine expression in patients with COVID-19 (*10*). CSS, while not specifically defined, is clearly observed in conditions such as hemophagocytic lymphohistiocytosis (*11*), severe cases of avian influenza (*12*), Castleman disease (*13*), and in the cytokine release syndrome observed in a subset of patients following chimeric antigen receptor T cell therapy for malignancy (*14*). In these cases of true CSS, circulating blood levels of multiple cytokines are elevated usually more than 10-fold above baseline levels and generally demonstrate skewing toward a T helper 1 (T_H_1)–type cytokine response profile suggestive of an increased T cell response. Reported cytokine expression levels in studies of patients with COVID-19, although increased in individuals with severe illness, are elevated only marginally, less than twofold above those observed in noncritically ill subjects (*3*). Very few studies compare COVID-19 inflammatory responses to those observed in other viral illnesses that lead to acute respiratory failure, such as influenza (*15*).

There has been significant interest in modulating the systemic immune response in an effort to prevent or treat respiratory failure in patients with COVID-19 (*2*, *8*, *16*–*18*). More than 100 clinical trials are currently registered at clinicaltrials.gov to evaluate the efficacy of inflammatory cytokine blocking medications or interventions such as cytokine filtration as potential treatments for respiratory failure in patients with COVID-19. The RECOVERY dexamethasone study suggests a mortality benefit for immunomodulation therapy in a subset of critically ill patients with COVID-19 (*8*). A thorough understanding of the underlying inflammatory environment in patients with COVID-19 is required to carefully select patients for these studies of potential treatments, especially in light of the increased (although not statistically significant) rate of death observed in patients without an oxygen requirement who were given dexamethasone in the RECOVERY trial.

To understand the relationship between inflammatory host responses and the respiratory failure observed in patients with COVID-19, we undertook a comparative investigation of inflammatory responses in a cohort of influenza patients with severe illness collected during 2019–2020, which allowed us to characterize the immune response in patients with severe COVID-19 specifically in the context of the more widely studied immune responses seen in respiratory disease caused by influenza. These comparisons reveal that, despite prevailing assumptions in the literature, CSS is relatively rare among moderate and severe COVID-19 infections; rather, most patients with COVID-19 in the current study exhibited suppressed immune profiles relative to the patterns observed among influenza-infected patients.

## RESULTS

### Demographic and clinical characteristics

We enrolled a total of 79 symptomatic subjects in the initial (primary) cohort who tested positive for SARS-CoV-2 RNA using a Food and Drug Administration–approved clinical polymerase chain reaction (PCR) test. Our comparison group consisted of 26 symptomatic seasonal influenza subjects recruited during the period of 15 months immediately preceding the outbreak of COVID-19 in the Saint Louis region, all of whom tested positive for influenza A or B via a clinical PCR test obtained during their clinical care. COVID-19 subjects were, on average, 19 years older than influenza subjects and 29 years older than control subjects (Table 1). A greater number of COVID-19 subjects required hospitalization, ICU admission, and mechanical ventilation than influenza subjects, but this was not significantly different after controlling for demographic factors and other clinical characteristics. Twenty-seven percent of the COVID-19 subjects died during their hospitalization compared with 8% of influenza subjects enrolled. Many subjects in both influenza and COVID-19 groups exhibited comorbidities that increased their risk for severe disease, including diabetes and chronic lung disease; however, there were no significant differences between the COVID-19 and influenza subjects in any analyzed comorbidity (Table 1). Both the COVID-19 and influenza cohorts included subjects with moderate disease, as defined by individuals with symptomatic illness requiring evaluation in the hospital, and severe disease, as defined by individuals requiring mechanical ventilation for acute respiratory failure or who ultimately died due to their illness.

**Table 1:** Demographics and clinical characteristics of primary cohort. Statistical analyses of primary cohort demographics include comparisons between COVID-19 and healthy groups for age, sex, and ethnicity using a multinomial logistic regression that includes the variation from influenza-infected individuals. Comparisons between COVID-19 and influenza groups were assessed using a multivariate logistic regression that included demographic and clinical variables but without including variation from the healthy group, as clinical characteristics were irrelevant to healthy controls. Results are reported as the corresponding *P* value and, when significant, include the odds ratio (OR). COVID-19 serves as the reference condition in all analyses, “African American” serves as the reference for ethnicity comparisons, and the negative indication serves as the reference for all categorical clinical characteristics. A significant OR > 1 indicates a positive association between the comparator group and the variable (e.g., healthy individuals were more likely to self-identify as Caucasian than patients with SARS-CoV-2), whereas a significant OR < 1 indicates a negative association (e.g., influenza-infected patients were likely to be younger than patients with SARS-CoV-2). The “immunocompromised” comorbidity was not included in the logistic regression due to complete separation across conditions and was instead tested using Fisher’s exact test. *P* values were adjusted for multiple testing by controlling the false discovery rate. SD denotes standard deviation, and IQR denotes interquartile range. N.S., not significant.

	**SARS-CoV-2** **(*n* = 79)**	**Healthy** **control** **(*n* = 16)**	**Influenza** **(*n* = 26)**	**COVID-19–healthy** **comparison**	**COVID-19–** **influenza** **comparison**
**Demographics**					
Means ± SD (range) age, in years	61 ± 15 (25–89)	32 ± 7 (22–49)	42 ± 17 (18–89)	*P* < 0.001, OR = 0.85	*P* = 0.007, OR = 0.93
Female	44% (35/44)	50% (8/8)	58% (15/26)	*P* = 1, N.S.	*P* = 1, N.S.
**Ethnicity**					
African American	80% (63/79)	44% (7/16)	65% (17/26)	–	–
White	18% (14/79)	56% (9/16)	27% (7/26)	*P* < 0.05, OR = 9.59	*P* = 0.718, N.S.
Other	<3% (2/79)	0% (0/16)	8% (2/26)	–	*P* = 1, N.S.
**Clinical characteristics**					
Mean (IQR) symptom duration at study enrollment, in days	6.4 (3–9)		4.1 (2–7)		*P* = 0.229, N.S.
Hospital admission	90% (71/79)		58% (15/26)		*P* = 0.229, N.S.
ICU admission	56% (44/79)		35% (9/26)		*P* = 0.285, N.S.
Intubation and mechanical ventilation	44% (35/79)		27% (7/26)		*P* = 0.285, N.S.
In-hospital death	30% (24/79)		8% (2/26)		*P* = 0.234, N.S.
**Comorbidities**					
Immunocompromised	6% (5/79)		0% (0/26)		*P* = 0.33, N.S.
Chronic lung disease	34% (27/79)		42% (11/26)		*P* = 0.682, N.S.
Chronic heart failure	13% (10/79)		23% (6/26)		*P* = 0.101, N.S.
End-stage renal failure	5% (4/79)		8% (2/26)		*P* = 0.582, N.S.
Diabetes mellitus	43% (34/79)		27% (7/26)		*P* = 0.628, N.S.
Active cancer	6% (5/79)		8% (2/26)		*P* = 0.234, N.S.

### Evaluation of circulating immune cells

**Fig. 1:**
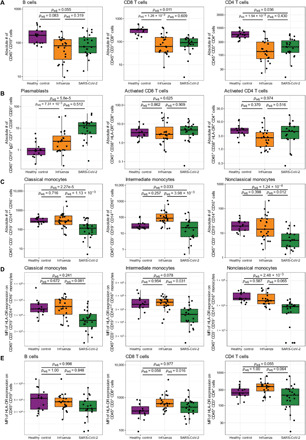
Evaluation of circulating lymphocyte and monocyte subpopulations in select healthy controls (*N* = 15), acute influenza-infected subjects (*N* = 23), and acute SARS-CoV-2–infected subjects (*N* = 22). Absolute numbers of (**A**) B cells, CD8^+^ T cells, and CD4^+^ T cells; (**B**) circulating B cell plasmablasts, activated CD8^+^ T cells, and activated CD4^+^ T cells; and (**C**) classical, intermediate, and nonclassical monocytes were quantified by flow cytometry. Surface expression of the major histocompatibility complex class 2 molecule, HLA-DR, on the surface of the indicated subpopulations of circulating monocytes (**D**) and lymphocytes (**E**) as measured by geometric mean fluorescence intensity (MFI) using flow cytometry. Presented *P* values are from pairwise comparisons of estimated marginal means of linear regression models that adjust for ethnicity, sex, age, and all comorbidities (immunocompromised, end-stage renal disease, chronic lung disease, chronic heart failure, and diabetes mellitus). *P* values were adjusted for multiple comparisons using Tukey’s method. For comparisons between COVID-19 and influenza, the models also include days of symptom duration at study enrollment as a covariate. In each case, raw values are plotted on the log10 scale.

Using peripheral blood mononuclear cells (PBMCs) from 15 healthy, 23 influenza-infected, and 22 COVID-19–infected subjects, we examined the composition and activation of circulating leukocytes with flow cytometry. We used multivariate linear regression with subject age, sex, ethnicity, symptom duration at study enrollment, and all comorbidities as covariates to explore immune cell dynamics as a function of condition while statistically controlling for demographic and other clinical differences across the patient groups. We initially characterized circulating immune cells by quantifying the absolute number of CD4^+^ and CD8^+^ T lymphocytes and CD19^+^ B cells. COVID-19 and influenza subjects exhibited trends of decreased B cells and significant reductions in both T cell subsets, which generally constitute most of the circulating PBMCs in healthy controls (Fig. 1A and fig. S1A). In contrast, COVID-19 subjects had significantly more circulating early antibody-secreting B cell plasmablasts than controls (Fig. 1B). Circulating activated CD4^+^ and CD8^+^ cells were equivalent across all groups (Fig. 1B). However, when compared with either influenza or control subjects, COVID-19 subjects exhibited significantly reduced numbers of circulating monocytes, including all three common classifications of human monocytes (classical, intermediate, and nonclassical; Fig. 1C and fig S1B).

Given the pronounced variation in monocyte abundance across patient conditions, we also measured major histocompatibility complex class II expression on the surface of monocytes to gauge monocyte activation. We noted that COVID-19 subjects had reduced abundances of HLA-DR on the surface of all classes of monocyte when compared with influenza subjects or controls, although only intermediate monocytes reached statistical significance after controlling for covariate effects (Fig. 1D). In addition, patients with COVID-19 exhibited significantly less surface HLA-DR on CD8^+^ T cells than patients with influenza and trends toward less HLA-DR on CD4^+^ T cells in comparison to both patients with influenza and healthy controls (Fig. 1E). Once again using multivariate linear regression, we next assessed potential differences in HLA-DR abundance between patients with moderate illness and those with severe illness, defined as those who required intubation and mechanical ventilation or who ultimately expired as a result of their illness. Although there were no associations with severity in HLA-DR abundance among lymphocyte populations, we found that, compared to moderately ill patients, the severest patients exhibited substantially less HLA-DR on intermediate and nonclassical monocytes (fig. S1, C and D).

### Cytokine associations with disease

**Fig. 2:**
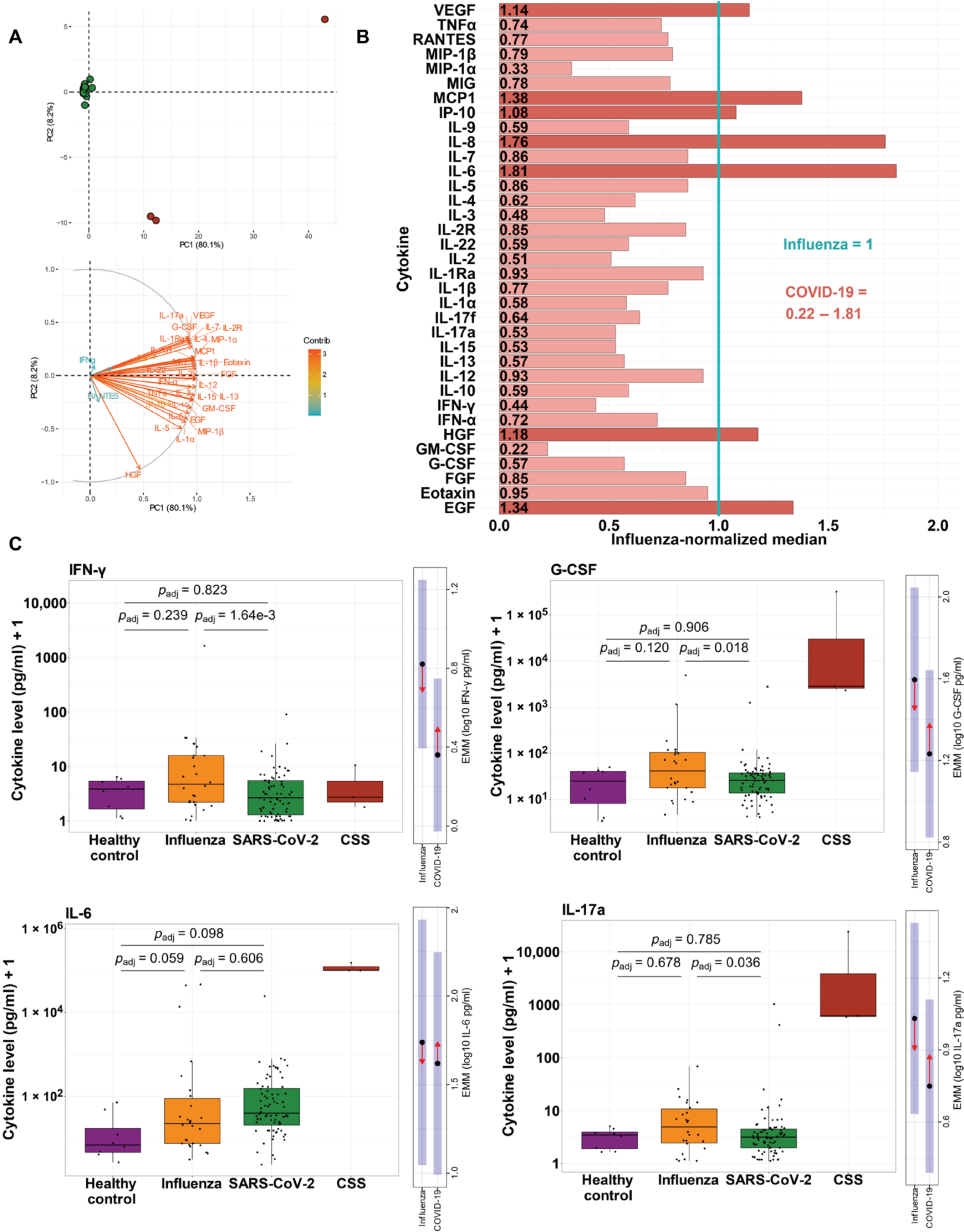
Selective cytokine up-regulation in patients with COVID-19 from the primary cohort. (**A**) Top: PCA of 35 cytokines measured in COVID-19 subjects from the primary cohort. Red circles: patients with CSS; green dots: all other subjects. Samples with missing cytokine data were excluded. Bottom: corresponding PCA loadings indicating effects of each cytokine. (**B**) Relative cytokine abundance plot, with each cytokine normalized to the respective median cytokine level in influenza subjects. The normalized median cytokine level in influenza patients (1.0) is represented by the vertical blue line. Bar graphs represent the normalized median COVID-19 cytokine level relative to the normalized median influenza cytokine level. Light red bars: cytokine levels lower in COVID-19 than influenza patients (normalized median < 1, *n* = 28); dark red bars: cytokines levels greater in patients with COVID-19 than in patients with influenza (normalized median > 1, *n* = 7). (**C**) Box plots show cytokine concentrations in healthy, influenza, COVID-19, and CSS subjects, with raw values plotted on the log10 scale. *P* values are from estimated marginal means (EMM) comparisons, averaging over all demographic and clinical factors included as covariates and adjusted for multiple comparisons. To the right of each box plot are EMM plots for the influenza–COVID-19 comparison. Black dot: estimated marginal mean for the log10 concentration of the cytokine, averaged over the levels of all other covariates; blue shading: corresponding 95% confidence interval; red arrows: SE in one direction, with overlapping SE arrows indicating no significant difference between the EMM of a given cytokine in influenza subjects versus COVID-19 subjects.

From this primary cohort, plasma cytokine levels were measured from 79 patients with SARS-CoV-2 (COVID-19) infection, 26 patients with confirmed influenza virus infection, and 8 healthy controls. Among the patients with COVID-19, two response profiles were immediately apparent, with 3 of 79 patient samples exhibiting obviously distinct cytokine profiles in principal components analysis (PCA; Fig. 2A). These samples were characterized by cytokine levels of >2 SDs from the mean for more than 17 of the 35 cytokines measured (range: 49 to 89%), encompassing broad and unfocused immune responses characteristic of classic cytokine storm (see outliers; fig. S2). Cytokine storms in other conditions have been defined by extreme deviations in the levels of a broad array of cytokines rather than just moderate elevations in targeted pathways (*19*). SDs from the mean ranged from 2 to 10.5 among these CSS subjects, with outlier values ranging from 0.8 to 2 orders of magnitude higher than the mean for each of the measured cytokines. Patients with CSS were all African American, one female (89 years) with no noted comorbidities, one female (62 years) with diabetes mellitus, and a male (47 years) with diabetes mellitus and preexisting chronic pulmonary disease (table S1). All three patients with CSS were admitted to the ICU, required intubation and mechanical ventilation, and ultimately expired. In subsequent analyses, these three patients were visualized and analyzed as a separate CSS group unless otherwise noted. Among the remaining 76 COVID-19 samples from this primary cohort, patients often exhibited values outside 1.5 interquartile ranges for individual cytokines (fig. S3), suggestive of activation of specific pathways; however, the vast majority of each patient’s individual cytokine levels were well within the majority of the observed variation, which does not support the broad dysregulation of cytokines expected in a CSS phenotype.

We and others have previously shown that many cytokines are often correlated with demographic and environmental factors (e.g., age and prior herpesvirus exposure) (*20*). To assess cytokine differences across groups while controlling for these potentially confounding factors, including the significant differences in age observed within our cohort, we generated estimated marginal means from linear regression models that incorporated age, sex, ethnicity, days since symptom onset at enrollment, and all reported comorbidities as covariates. In comparison to healthy controls, both influenza and COVID-19 subjects exhibited elevated levels of a number of cytokines. Among patients with COVID-19, IP-10, IL-8, MCP1, HGF (hepatocyte growth factor) and MIP-1β were significantly up-regulated compared to healthy controls, in addition to apparent (but not statistically significant) trends for increases in MIG (monokine induced by interferon gamma), granulocyte-macrophage CSF (GM-CSF), IL-1RA, IL-2, IL-17f, and IL-6 (Fig. 2B and fig. S3). In comparison to healthy controls, influenza-infected patients likewise exhibited significant up-regulation of all of cytokines up-regulated among patients with COVID-19, but influenza-infected patients also exhibited significantly greater abundances (compared to COVID-19–infected patients) of a number of cytokines with known inflammatory and immunomodulatory roles, including MIG, IL-1RA, IL-2R, IL-2, IL-17f, and IL-12 (fig. S3).

To elucidate the unique ways in which COVID-19 modulates cytokine profiles, we focused the majority of cytokine analyses on comparisons between the COVID-19 and influenza groups. Direct comparisons between infected groups revealed that the dominant response profile among patients with COVID-19 consisted of more selective cytokine up-regulation, with a relative bias toward lower inflammation when compared to patients with influenza (Fig. 2, B and C). Among all subjects, we found that for 28 of 35 cytokines, patients with COVID-19 had lower median cytokine levels, although not all were statistically significant (Fig. 2B and fig. S3). Among the statistically significant reduced cytokines exhibited by patients with COVID-19 compared to influenza patients were interferon-γ (IFN-γ), MIG, IL-1RA, IL-2R, G-CSF, IL-17a, IL-9, and MIP-1α. Upon visual inspection of the raw data, both IL-6 and IL-8 appeared to be greater among COVID-19 than influenza-infected patients, but the estimated marginal means of neither cytokine was significantly different after controlling for covariates (Fig. 2C and figs. S3 and S4), particularly the effects of age, sex, and preexisting pulmonary disease. This finding is particularly relevant in the ongoing discussion of COVID-19 cytokine profiles: Although some cytokines seem to be higher among patients with COVID-19, those differences are potentially inflated by other underlying demographic and clinical differences. For instance, hospitalized patients with COVID-19 are generally older [Table 1; (*21*)], and many baseline cytokine levels increase with age (*22*, *23*). When accounting for these and other confounding factors, the data indicated that most patients with COVID-19 did not have an inflammatory phenotype as profound as patients with influenza, with certain targeted exceptions.

### Validation of COVID-19 cytokine patterns in an additional SARS-CoV-2 cohort

**Fig. 3:**
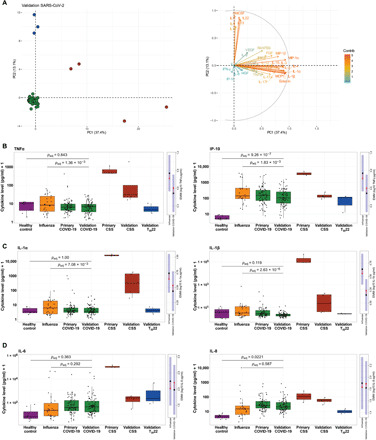
Selective cytokine up-regulation in COVID-19–infected patients from the validation cohort. (**A**) Left: PCA of all 35 cytokines measured in the COVID-19 validation cohort. Red circles represent samples with CSS cytokine profiles, blue dots represent samples with T_H_22 cytokine profiles, and green dots represent the remainder of subjects. Samples with missing cytokine data were excluded. Right: The corresponding PCA loading plot, in which each cytokine has a vector/arrow which points in the direction of increasing cytokine levels. The color and length of the vector represent how strongly each cytokine contributes to a principal component. (**B** to **D**) Box plots show cytokine concentrations in healthy, influenza, primary COVID-19, validation COVID-19, primary CSS, validation CSS, and validation T_H_22 groups for all 35 cytokines measured, with raw values plotted on the log10 scale. Presented *P* values are from estimated marginal means (EMM) comparisons, averaging over all demographic and clinical factors that were included as covariates and *P* values adjusted for multiple comparisons. Although T_H_22 samples were visualized separately, they were included as validation COVID-19 samples in the underlying statistical analyses. To the right of each box plot are EMM plots for the influenza–COVID-19 comparison; the black dot represents the estimated marginal mean for the log10 concentration of the cytokine for a given condition, averaged over the levels of all other covariates (e.g., age, sex, and ethnicity), and the blue shading represents the corresponding 95% confidence interval. The red arrows represent the SE in one direction, with overlapping SE arrows indicating no significant difference between the EMM of a given cytokine in influenza subjects versus COVID-19 subjects.

To validate the cytokine patterns observed among COVID-19, influenza, and healthy control groups in our primary cohort, we enrolled a follow-up cohort that consisted of the next 89 consecutive confirmed patients with COVID-19 enrolled into the ongoing prospective observational study (Table 2). Cytokine data from these patients were collected and analyzed as described for the primary cohort. Among the 89 patients in the validation cohort, four exhibited marked variation in their cytokine profiles (Fig. 3A, red circles) that, although not as extreme as those in the primary cohort, were consistent with a CSS phenotype (fig. S5). These samples were characterized by cytokine levels of >2 SDs from the mean for more than 9 of the 35 cytokines measured (range: 26 to 49%). Two of these patients self-identified as African American (59-year-old female, 71-year-old male), one as “other” (41-year-old male), and one as white (64-year-old male). Both African-American patients with CSS had been previously diagnosed with diabetes mellitus, and the 59-year-old female also had been diagnosed with chronic heart failure and chronic pulmonary disease and was immunosuppressed. The other patients with CSS had no listed comorbidities. The 64-year-old male patient was admitted to the ICU, required intubation and mechanical ventilation, and ultimately expired. None of the other three patients with CSS were intubated, and all ultimately survived. In contrast to these CSS profiles, another subset of three patients exhibited a unique profile with a classic T_H_22 signature (Fig. 3A, blue circles), consisting of production of high levels of IL-22, GM-CSF, and IL-13 without high levels of IL-17. All patients with a T_H_22 signature were female African Americans (aged 19, 26, and 71) with no listed comorbidities. None required intubation, and all survived. Although samples exhibiting the T_H_22 signature are plotted separately for visual comparison, they were considered to represent regulated cytokine responses and were therefore included as COVID-19 validation samples in all statistical analyses.

**Table 2:** Demographics and clinical characteristics of validation cohort with comparisons to primary cohort subsets. Statistical analyses of validation cohort demographics include comparisons between validation and healthy groups for age, sex, and ethnicity using a multinomial logistic regression that includes the variation from influenza-infected individuals. Comparisons between the validation cohort and both patient groups from the primary cohort (COVID-19 and influenza) were assessed using a multivariate logistic regression that included demographic and clinical variables. Results are reported as the corresponding *P* value and, when significant, include the OR. The validation cohort serves as the reference condition in all analyses, African American serves as the reference for ethnicity comparisons, and the negative indication serves as the reference for all categorical clinical characteristics. A significant OR > 1 indicates a positive association between the comparator group and the variable (e.g., patients with COVID-19 from the primary cohort were more likely to be intubated than validation cohort patients with COVID-19), whereas a significant OR < 1 indicates a negative association (e.g., healthy controls were likely to be younger than validation cohort patients). *P* values were adjusted for multiple testing by controlling the false discovery rate. SD denotes standard deviation, and IQR denotes interquartile range. The “immunocompromised” comorbidity was not included in the validation-influenza comparison logistic regression due to complete separation across groups and was instead tested using Fisher’s exact test.

	**SARS-CoV-2 validation** **(*n* = 89)**	**Validation-primary** **COVID-19 comparison**	**Validation-healthy** **comparison**	**Validation-influenza** **comparison**
**Demographics**				
Means ± SD (range) age, in years	61 ± 17 (19–92)	*P* = 1, N.S.	*P* < 0.001, OR = 0.87	*P* = 0.041, OR = 0.93
Female	39% (35/89)	*P* = 1, N.S.	*P* = 1, *N.S.*	*P* = 1, N.S.
**Ethnicity**				
African American	74% (66/89)	–	–	–
White	25% (22/89)	*P* = 1, N.S.	*P* = 0.069, *N.S.*	*P* = 1, N.S.
Other	1% (1/89)	*P* = 1, N.S.	–	*P* = 1, N.S.
**Clinical characteristics**				
Mean (IQR) body mass index	28.6 (24–33)	–		–
Mean (IQR) symptom duration at study enrollment, in days	7.5 (2–9)	*P* = 0.958, N.S.		*P* = 0.635, N.S.
Hospital admission	94% (84/89)	*P* = 0.958, N.S.		*P* = 0.043, OR = 0.046
ICU admission	48% (43/89)	*P* = 0.10, N.S.		*P* = 1, N.S.
Intubation and mechanical ventilation	27% (24/89)	*P* < 0.001, OR = 19.03		*P* = 0.893, N.S.
In-hospital death	17% (15/89)	*P* = 0.509, N.S.		*P* = 1, N.S.
**Comorbidities**				
Immunocompromised	8% (7/89)	*P* = 1, N.S.		*P* = 0.347, N.S.
Chronic lung disease	16% (14/89)	*P* = 0.017, OR = 5.9		*P* = 0.049, OR = 11.69
Chronic heart failure	15% (13/89)	*P* = 1, N.S.		*P* = 0.817, N.S.
End-stage renal failure	2% (2/89)	*P* = 1, N.S.		*P* = 1, N.S.
Diabetes mellitus	44% (39/89)	*P* = 1, N.S.		*P* = 1, N.S.
Active cancer	3% (3/89)	*P* = 0.893, N.S.		*P* = 0.387, N.S.

Comparison of cytokine measures from the validation cohort to the original healthy controls and patients with influenza were largely consistent with those observed in the primary cohort. In addition to significantly higher levels of IP-10, IL-8, HGF, and MIP-1β observed among patients with COVID-19 from the primary cohort in comparison to healthy controls, the validation cohort also exhibited significantly greater levels of IL-12, epidermal growth factor (EGF), and IL-2 (fig. S6), two of which were consistent with trends observed in the primary cohort analyses (fig. S3).

In comparison to influenza-infected subjects, the COVID-19 subjects in the validation cohort also exhibited many of the same patterns observed within the primary COVID-19 cohort with the single exception of MIP-1α (*P* = 0.14), which may have differed due to variations in the assay’s lower limits of detection. In addition, the validation COVID-19 cohort in comparison to influenza-infected subjects exhibited significantly lower levels of IL-1β, IL-4, IP-10, TNFα, IL-1α, IL-17f, fibroblast growth factor (FGF), and eotaxin (Fig. 3, B and C, and figs. S6 and S7), several of which were consistent with nonsignificant trends observed in the primary cohort analyses (figs. S3 and S4). We again detected no significant difference in either IL-6 or IL-8 between influenza-infected and COVID-19–infected patients after controlling for demographic and clinical covariates (Fig. 3D). Overall, these data validated our observation in the primary cohort that many cytokines are down-regulated in patients with COVID-19 compared to influenza-infected patients, suggesting that overall higher inflammation is foremost predictive of influenza infection and that a defining feature of COVID-19 disease is generally reduced inflammation compared to influenza; aside from the 4% of patients with extreme cytokine dysregulation (i.e., CSS), COVID-19 subjects were not characterized by overall high levels of cytokines but rather exhibited a selective pattern of inflammation in which only a subset of inflammatory cytokines were up-regulated, and most were down-regulated when compared with seasonal influenza patients.

### Cross-cohort comparisons and integrated cytokine analyses

Comparison of the validation cohort to the COVID-19 group from the primary cohort revealed no significant differences in demographics or comorbidities, save for a significant reduction in preexisting chronic lung disease (16% in the validation cohort compared to 34% in the original cohort; Table 2). Of the other clinical characteristics considered, the validation cohort was significantly less likely to receive mechanical ventilation (27% compared to 44%). We hypothesize that this difference reflects the evolution of treatment approaches over time rather than an underlying difference in the patient population, as the difference in ventilation rates was significant after controlling for differences in comorbidities, and there was no significant difference in death rates between the cohorts. Furthermore, written guidance to the clinical staff at the study institutions over the course of the first weeks of the pandemic reflected a nationwide transition from initial recommendations for early and aggressive intubation of patients with COVID-19 with relatively moderate hypoxia to later written guidance that focused on maximizing oxygen delivery strategies before intubation using more traditional approaches to acute respiratory failure. Although we found a significant negative association between the week of enrollment and the percentage of patients intubated (fig. S8A), we observed the opposite relationship between the week of enrollment and the percentage of patients who survived infection (fig. S8B). These correlations suggest that aggressive early intubation strategies may have been unwarranted or even harmful, but further study is required. An alternative hypothesis is that disease severity somehow naturally declined over time among patients with COVID-19 seeking hospital care in the St. Louis area.

**Fig. 4:**
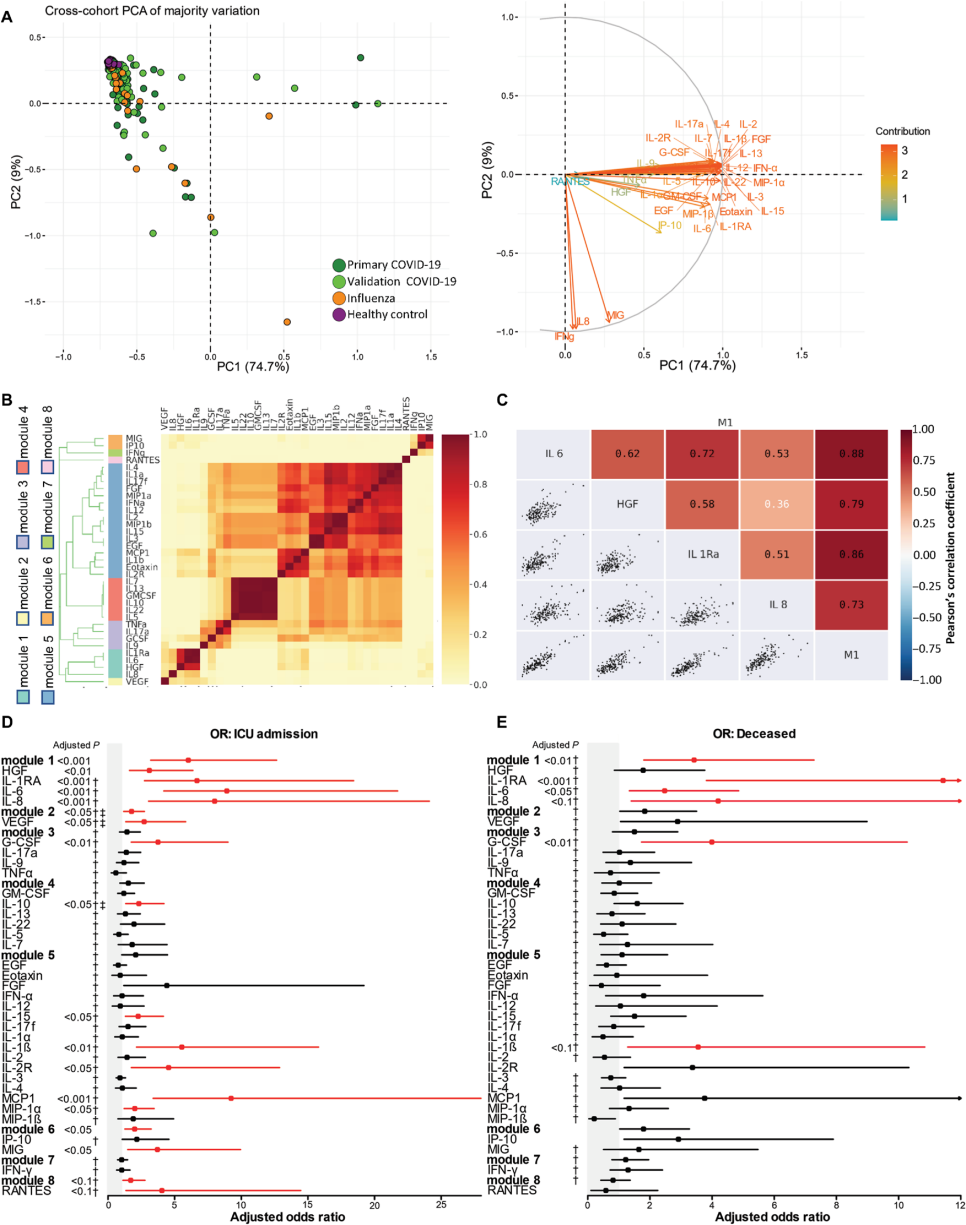
Cross-cohort comparisons. (**A**) Left: PCA of 35 cytokines measured across all patient groups in both primary and validation cohorts; all samples with complete cytokine data were included in the analysis, but only the majority of sample variation is included in the visualization. Right: corresponding PCA loadings indicating effects of each cytokine. (**B**) Correlations of log10 absolute cytokine values are visualized as the proportion of times cytokine pairs clustered together during hierarchical clustering over 1,000 permutations for all COVID-19 patients across cohorts. Colors alongside the dendrogram and cytokine names denote module membership, whereas colors within the heatmap correspond to the ratio of coclustering. (**C**) Cytokine cocorrelations for all cytokines assigned to Module 1 (M1) are assessed using Pearson’s correlation coefficient. (**D** and **E**) Forest plots depicting the adjusted odds ratios obtained from multivariate logistic regression analysis between cytokines and various correlates of COVID-19 disease severity. (D) Cytokine associations with ICU admission. (E) Cytokine associations with death. Logistic regression models used absolute log10-transformed cytokine values and included age, sex, ethnicity, days since symptom onset at enrollment, all reported comorbidities, and cohort as covariates. Gray shading indicates the area of the plots where odds ratios are less than 1, indicative of negative associations. Adjusted odds ratios are indicated with points, and confidence lines encompass the range between the lower and upper limits. Red indicates significance at false discovery rate < 0.1, † indicates that age was a significant covariate, and ‡ indicates that day of sampling after symptom onset was significant.

Regardless of these differences, many of the underlying cytokine patterns were replicated between the primary and validation cohorts, as most of the cytokine variation among the two COVID-19 cohorts overlapped substantially (e.g., Fig. 4A and fig. S8, C and D). Yet, other patterns seemed not to replicate to statistical significance simply due to a lack of power for the highly parameterized models required to control for all demographic and clinical factors (*20*). This was not particularly unexpected as the relatively high dimensionality of cytokine data and the tendency for extreme outliers can make it difficult to detect statistically significant associations while conservatively controlling for false discovery rate. To address this, we next sought to integrate the cytokine data from the two COVID-19 cohorts by using a data-driven modular informatics approach developed specifically for cytokine analyses (*24*). These analyses allowed us to detect a number of co-correlating cytokines across COVID-19 samples, which we grouped into distinct coexpression modules using hierarchical clustering (Fig. 4B). This unsupervised approach categorized the 35 cytokines we assayed among COVID-19 samples into eight distinct modules of cosignaling cytokines (fig. S8E), including a module comprising HGF, IL-1RA, IL-6, and IL-8 (module 1; Fig. 4C). As described above, most elements of this module were independently found to be up-regulated in both influenza and COVID-19 groups compared to healthy controls and were comparably expressed between COVID-19 and influenza groups, save for IL-1RA, which showed decreased expression among COVID-19 subjects compared to influenza subjects. Plasma IL-6 and IL-8 have been shown to correlate strongly in large pediatric and adult influenza cohorts, albeit typically with G-CSF and MIP-1α as well (*24*); in contrast, among COVID-19 samples, G-CSF clustered with IL-1α, IL-9, and TNFα (module 3), whereas MIP-1α clustered with 13 other cytokines, including several ILs and IFN-α (module 5). Module 5 contained cytokines associated with type 1 (IL-12), type 2 (IL-4), and type 3 (IL-17f) immune responses, including those secreted by innate (IFN-α and IL-1) and adaptive (IL-4 and IL-15) immune cells. Notably, several cytokines were assigned to their own modules due to a lack of sufficient correlation with others, including the chemokine RANTES (module 8), vascular endothelial growth factor (VEGF; module 2), and IFN-γ (module 7). In general, these analyses suggest that aspects of typical cytokine cosignaling modules inferred from large cohorts in other severe respiratory diseases are altered in the context of COVID-19, indicative of a unique immunoregulatory environment in COVID-19 potentially demonstrated by the overall reduced inflammatory profile. The significantly lower levels of IFN-γ and its lack of clustering with other cytokines suggests that this prototypical type 1 cytokine is not being produced in a manner typical of other common viral infections.

Using this analytical framework further, we next inquired whether particular cosignaling modules or individual cytokine expression patterns were associated with clinical outcomes among patients with COVID-19. Here, we used multivariate logistic regression, again controlling for age, sex, ethnicity, days since symptom onset at enrollment, and all reported comorbidities to calculate adjusted odds ratios of severity. Because these analyses spanned both primary and validation cohorts, we also included cohort as a covariate to control for any potential cryptic differences between the patient populations, and we adjusted for multiple comparisons (13 covariates tested within each model) by controlling the false discovery rate. These analyses revealed a number of significant associations between various measures of disease severity, cosignaling cytokine modules, and their constituent cytokines. For instance, increases in cytokine modules 1, 2, and 6 were associated with increased odds of requiring ICU admission, as were HGF, IL-1RA, IL-6, IL-8, VEGF, G-CSF, IL-15, IL-1β, MCP1, MIP-1α, and MIG individually (Fig. 4D). Increases in module 1 and most of its constituent cytokines, as well as G-CSF and IL-1β, were likewise associated with increased risk of death (Fig. 4E). While some of these cytokines (IL-6 and IL-8) have been implicated previously in COVID-19 pathogenesis, our comprehensive screening approach has identified a signature that is notable for its focus on largely innate inflammatory mediators associated with monocyte and neutrophil mobilization. In addition, in our analysis, there was a lack of association with traditional adaptive inflammatory markers (IFN-γ, IL-13, and IL-5) that has been observed in some other reports (*25*). Strikingly, this severity signature is reminiscent of the innate monocyte mobilization signature identified in patients with influenza using a similar statistical approach, with the notable lack of IFN-α associations (*20*, *24*).

### Single-cell transcriptional profiles of COVID-19 subjects with respiratory failure are concordant with signals of targeted immunosuppression

**Fig. 5:**
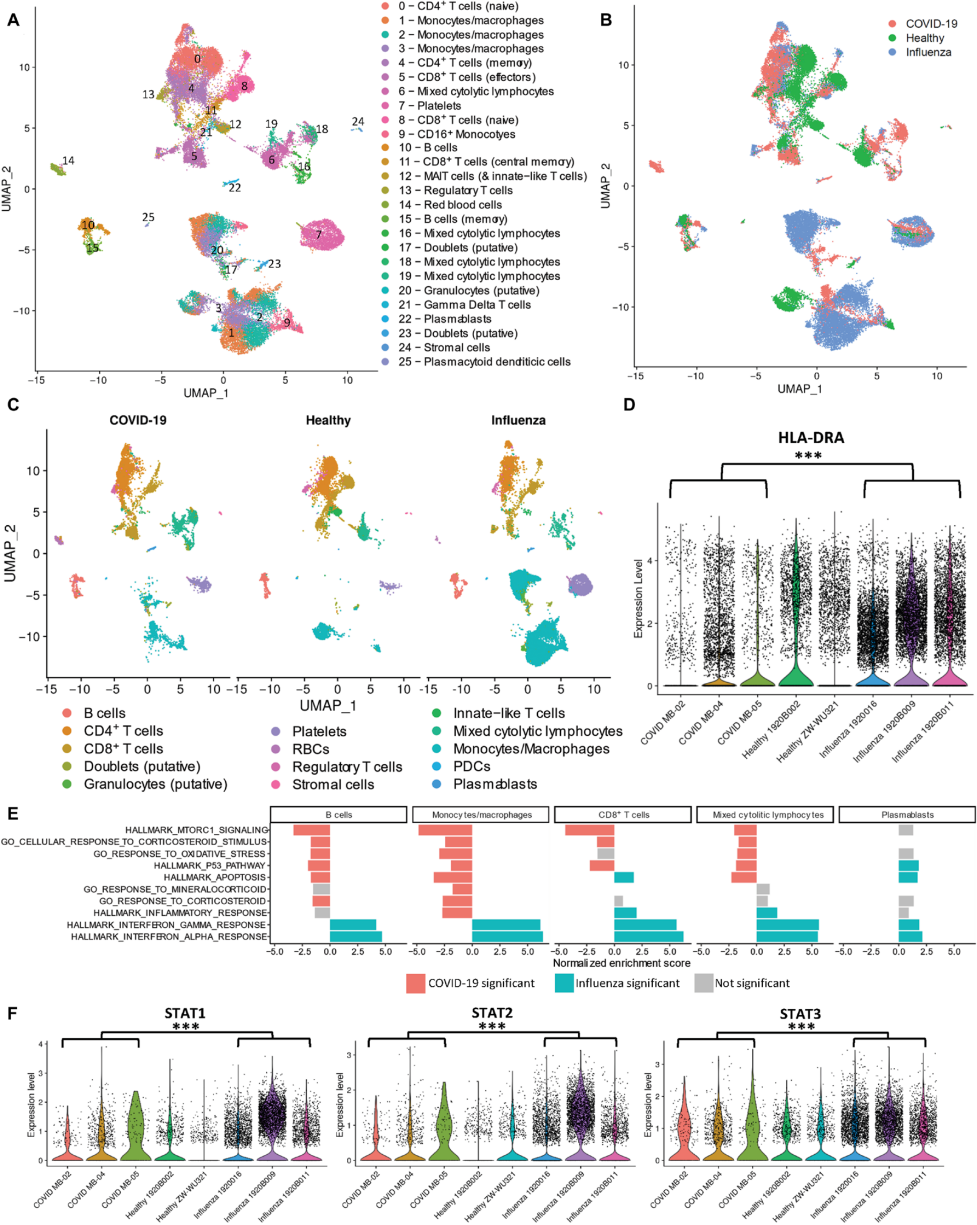
Single-cell gene expression analyses of PBMCs from COVID-19–infected, influenza-infected, and healthy participants demonstrate profound differences in the relative abundance and transcriptional activity of cell subsets across conditions. (**A**) Uniform Manifold Approximation and Projection (UMAP) plots depict transcriptional clusters, which (**B**) vary transcriptionally as a function of condition despite the presence of nearly all subsets across various conditions, as evidenced in (**C**). (**D**) Violin plots demonstrate significant down-regulation of HLA-DRA among all cells from COVID-19–infected patients compared to influenza-infected patients (with healthy controls included for reference). Asterisks indicate significance at Bonferroni-corrected *P* values < 0.001. (**E**) GSEA analysis of gene expression differences between COVID-19 and influenza groups across major cell subsets. In direct comparison to cells from influenza-infected patients, transcriptional patterns among cells from COVID-19–infected patients reveal significant up-regulation (red bars) of metabolic pathways, stress pathways, and glucocorticoid signaling pathways across major cell subsets, particularly monocytes/macrophages. In contrast, interferon pathways were significantly down-regulated (blue bars) among subsets from COVID-19–infected patients compared to those from influenza-infected patients. Gray bars indicate that tests for enrichment did not meet statistical significance for a particular subset. (**F**) Violin plots demonstrate significant down-regulation of STAT1, STAT2, and STAT3 among monocytes/macrophages from COVID-19–infected patients compared to influenza-infected patients (with healthy controls included for reference). Asterisks (***) indicate significance at Bonferroni-corrected *P* values of <0.001.

Immune suppression can often occur as a negative feedback from immune activation, so we sought further resolution of the immune state of a subset of severe COVID-19 subjects to understand the dominant regulatory signals determining their trajectory. A total of 37,469 cells from eight subjects (three COVID-19–infected, three influenza-infected, and two healthy controls) were obtained for single-cell gene expression analyses after standard processing and filtering. All six of the infected subjects required intubation and mechanical ventilation for severe respiratory failure, and ultimately three of the COVID-19–infected patients and one of the three influenza-infected patients died from their illnesses. Using an integration-based approach that leverages convergent expression signals across samples (see Materials and Methods), we identified 25 putative transcriptional clusters that we were able to categorize into major cell subsets, including monocytes and macrophages (four transcriptional clusters including CD16^+^ and CD16^−^ monocytes), CD8^+^ T cells (three clusters including putative naïve, effector, and central memory populations), CD4^+^ T cells (two clusters including putative naïve and memory populations), regulatory T cells (T_regs_), innate-like T cells [including gamma delta T cells and MAIT cells (mucosal-associated invariant T cells)], B cells (two clusters including putative memory B cells), plasmablasts, mixed cytolytic lymphocyte populations [MCLPs; four clusters that potentially include natural killer (NK) and NKT cells], platelets, red blood cells, granulocytes (putative), stromal cells, and plasmacytoid dendritic cells (PDCs), as well as putative doublets (Fig. 5A and fig. S9).

As variations in the relative abundance of subsets across groups were obvious upon visual inspection (Fig. 5, A to C), we next interrogated each of these major groups and their constituent transcriptional clusters for variation in both relative abundance and gene expression owed to differences in group (i.e., COVID-19–infected, influenza-infected, or healthy control). Although some transcriptional clusters had more cells from one condition or another (fig. S10), we used an analytical approach that allowed us to detect differences between conditions by simultaneously assessing the proportions of cells expressing a gene and the expression of that gene within cells expressing it.

Given the substantial down-regulation of HLA-DR among COVID-19 monocytes observed during flow cytometry analysis, we decided to further investigate potential transcriptional differences between COVID-19 and influenza specifically within our transcriptionally defined monocyte/macrophage subset and clusters. As expected, given the flow cytometry analysis carried out on these same patients, the proportion of cells in monocyte/macrophage clusters were substantially smaller in COVID-19 compared to both healthy and influenza subjects (fig. S10). In addition, expression of the conserved class II chain HLA-DR was significantly reduced in cells from patients with COVID-19 compared to patients with influenza (Fig. 5D).

At the single-cell transcriptional level, many of the genes that we profiled in our protein cytokine assays were rarely observed, likely due to the sensitivity limitations of the gene expression kit. We therefore used Gene Set Enrichment Analysis [GSEA; (*26*)] to broadly survey transcriptional variation as a function of infection status in an unbiased manner. Specifically, we ranked gene expression differences between COVID-19–infected and influenza-infected patients for each subset and tested for enrichment of Hallmark gene sets as a function of these diagnoses. Unexpectedly, a number of important immunological pathways were significantly enriched, specifically among cells from patients with influenza across a number of subsets: Compared to the influenza condition, both IFN-γ and IFN-α response pathways were significantly down-regulated within the COVID-19 condition for B cells, plasmablasts, CD8^+^ T cells, MCLPs, T_regs_, PDCs, and monocyte/macrophage subsets (Fig. 5E and fig. S11). These pathway-based analyses were particularly informative, as there was no significant difference in expression of IFN-γ itself in these subsets, and we were unable to detect IFN-α transcripts at all. More exhaustive analysis using gene ontology pathways related to interferon production, secretion, response, and regulation demonstrated that patterns observed among IFN-γ and IFN-α pathways extended to IFN-β (which was also not directly detected in the transcriptomic data) and general type I interferon pathways across most subsets but particularly among monocytes (fig. S11). These patterns were concordant with substantial enrichment of inflammatory pathways in influenza cells compared to COVID-19 cells across a majority of cell subsets. In contrast, COVID-19 cells were significantly enriched for a number of pathways involved in cellular metabolism, stress, corticosteroid stimulus, and proliferation in comparison to influenza cells across most subsets (Fig. 5E and figs. S12 and S13).

To confirm these observations, we looked at the specific expression of Stat genes, including the IFN-associated signal transducers and activators of transcription 1 (STAT1) and STAT2, which were both significantly underrepresented in patients with COVID-19 compared to patients with influenza (Fig. 5F). STAT3, which is critical for IL-6 signaling, was also expressed significantly less in patients with COVID-19 compared to patients with influenza despite the elevated levels of IL-6 circulating in these subjects. Together, these data indicate that, in this subset of patients with COVID-19, there was a general refractoriness to certain inflammatory signals, including IFNs. Although the transcriptional signals from this subset of patients were concordant with the robust phenotypic patterns observed across our much larger cohorts, it is important to note that others have reported dissimilar findings in their own transcriptional comparisons of treated influenza-infected and COVID-19–infected patients (*15*). Future work, as discussed below, will be important for fully understanding the diverse biological and immunological patterns underlying these heterogenous patient populations.

## DISCUSSION

Understanding the complexities of the systemic inflammatory response to SARS-CoV-2 infection is critical to determining the most appropriate treatment for this condition. We have demonstrated that the immunophenotypes of COVID-19 and influenza patients vary widely. Multiple forms of COVID-19 immune dysregulation were observed: a cytokine storm phenotype in 3 to 4% of patients across primary and validation cohorts (7 of 168), a T_H_22 phenotype in 3% of the validation cohort patients (3 of 89), and a far more common phenotype characterized by targeted immunosuppression relative to influenza-infected patients. The signatures of this common COVID-19 phenotype compared to influenza were equivalent levels of IL-6 and IL-8, paired with lower levels of cytokines in many other pathways and essentially the absence of any type I or type II IFN response. The suppression in type I IFN signaling has been noted by others in humans and animal models of COVID-19 infections (*27*–*29*). At the cellular level, marked reductions in overall cellularity, particularly in the monocyte compartment, were observed, with phenotypic and transcriptional evidence that monocytes were less activated. While lymphocyte numbers (except for plasmablasts) were reduced in both infected groups compared to healthy controls, several lymphocyte subsets had functional signatures of suppression in patients with COVID-19 compared to patients with influenza, including type I and II IFN signaling. IFN-γ production is critical for effector type I responses, and its absence may limit antiviral activity. The elevated plasmablast frequencies in COVID-19 patients may reflect the abundance of viral antigens, which is consistent with the reported persistence of viral RNA in nasal swabs for up to 15 days after onset of symptoms (*30*).

The single-cell analyses also identified enrichment of several pathways in COVID-19–infected patients associated with metabolic stress and the general stress response. These results, combined with the targeted, severe suppression of specific pathways and marked leuko- and lymphopenia, led us to consider what pathways might account for this response profile. Previous studies in animal models had implicated glucocorticoid (GC) signaling in the immunosuppression and lymphopenia that occurs in the influenza model in mice (*31*). In humans, systemic inflammation and hydrocortisone, specifically, are known to suppress HLA-DR expression on monocytes (*32*).

Excessive GC production is an attractive hypothesis to account for the observed immune dysregulation and disease manifestations in COVID-19. First, the relatively high levels of IL-6 production can directly drive excessive cortisol production through multiple mechanisms, including through the direct induction of corticotropin-releasing hormone and adrenocorticotropin. IL-6 can also act directly on the adrenal cortex to stimulate GC release (*33*). While GCs are generally immunosuppressive, which is why they are often used therapeutically, their effects are uneven across the cytokine landscape, with cortisol failing to suppress IL-6 (*34*) or even inducing IL-6 and IL-8 in one report (*35*). A recent retrospective cohort study from Germany has also reported increased cortisol levels in a majority of patients with COVID-19 (*36*), while another analysis found an association between risk of COVID-19 mortality and high levels of serum cortisol (*37*).

A recent report from the RECOVERY trial has suggested that, in contrast to this hypothesis, treatment with steroids (dexamethasone) had a significant protective effect in reducing mortality among the most severe COVID-19 patients (*8*). These data demonstrated a statistically significant reduction in mortality among patients requiring oxygen (21 versus 25%) or ventilation (29 versus 40%). In light of our findings, we would hypothesize that this subset of patients that was aided (~5 to 11% of the most severe patients and approximately 2% of the total cohort of COVID-19 patients enrolled in the study) represented those who had the severe cytokine storm immunotype. Intriguingly, in those patients not receiving respiratory support, outcomes for patients on dexamethasone were poorer, with 17% mortality versus 13.2% in the control group (although this was not statistically significant; *P* = 0.14). Other reports have similarly found deleterious effects of GC administration in COVID-19 and related illnesses (*38*). On the basis of our results, we might hypothesize that this lack of efficacy in this larger population may correspond to patients already experiencing higher levels of GC production. This hypothesis would need further verification, with direct measurement of GCs. If confirmed, therapeutic consideration should be given to inhibiting both IL-6 and GC activity in most of the patients with COVID-19 exhibiting this phenotype (high IL-6, low IFN signaling, and profound cytopenias) versus the small proportion of patients with a true cytokine storm phenotype. A focus on understanding GC dynamics and activity across the course of infection is an important direction for future research.

Cytokine storms were originally defined in graft versus host disease manifestations of hematopoietic stem cell transplants (*39*). Subsequent characterization of extraordinary levels of cytokines in H5N1 avian flu infections in vitro and in vivo led to an exploration of this phenotype in infection models (*40*). In general, seasonal influenza infections such as those studied here, even fatal cases were not associated with this phenotype, while the emergence of a previously unknown avian influenza virus (H7N9) again demonstrated a hypercytokinemia phenotype (*41*). Recent literature has started conflating “cytokine storms” with generally, but not exceptionally, elevated cytokine levels. We have tried to emphasize here that regardless of the terminology, this small but significant subgroup of patients with outlier levels of many cytokines needs special consideration and is most consistent with the “cytokine storm” literature (*11*, *13*, *42*). For instance, one patient with CSS had 1,080,000 pg/ml IL17f, whereas the average value among all patients with non-CSS COVID-19 is 1435 pg/ml.

Our findings are consistent with severe COVID-19 disease resulting from diverse underlying immunological profiles. While a subset of patients exhibit the cytokine storm phenotype, the majority do not, including most of those who progress to the severest outcomes. Another study using cell-based immune profiling also identified diverse “immunotypes” associated with varying outcomes, including strong activation and expansion in adaptive immune compartments (T cells and plasmablasts) in some patients, while other patients appeared to have no measurable adaptive immune activation above baseline (*43*). We attempted to define the cytokine storm subset using commonly measured clinical parameters (e.g., C-reactive protein levels), or combinations thereof, but thus far have not identified a statistically informative predictor. Other groups analyzing severe COVID-19 cohorts have similarly failed to directly correlate clinically measured parameters in patients with cytokine storm phenotypes (*44*). Ideally, parsing the diverse and therapeutically distinct immune profiles underlying severe COVID could be assessed with a simple, fast, inexpensive, robust test; a minimal set of cytokines (e.g., simultaneous assessment of some combination of EGF, FGF, IL-15, MIP-1α, IL-3, and/or other cytokines with consistent outliers in figs. S2 and S5) may currently be the most tractable option.

There are several important limitations to the current work, including our focus on the circulating peripheral blood compartment. At the initiation of the study, the safe collection of respiratory samples from patients with COVID-19 had not been established, although we plan to address the local features of inflammation in subsequent reports, including addressing whether some of the peripherally depleted subsets may have migrated to the inflamed airways. Ideally, longitudinal samples would also be used to determine the trajectory of each immune profile to determine their stability. In addition, we focused on the first blood sample collected from each subject, which, in more than 75% of enrolled subjects, was collected from the patient at the same time that the treating team learned of the patient’s COVID-19 diagnosis. Although limiting the possibility that substantial disease-specific therapeutic interventions were used before initial sample collection removed a major potential source of variation among our subjects, in future studies, it will be critical to understand how the array of therapeutic modalities currently being applied affect our identified immune profiles. Furthermore, our study compares initial enrollment blood samples from COVID-19 subjects and acute influenza subjects. While there was no significant difference between the duration of symptoms before sample collection between the two groups and multivariate analyses used throughout our manuscript controlled for duration of symptoms, there remain differences between these two viral infections; this may include the duration of the presymptomatic period of infection. The time course of the immune response to these two distinct viral pathogens is likely not identical despite our efforts to provide statistical correction for these variations. Last, our study does not include a truly mild COVID-19 group, such as asymptomatic patients. This group is particularly hard to enroll and sample in the current setting as they are requested to avoid the clinic, and it is important to note that our conclusions do not necessarily generalize to these milder infections.

## MATERIALS AND METHODS

### Study design

This is a prospective observational cohort of subjects with viral respiratory illness symptoms who presented to Barnes Jewish Hospital, St. Louis Children’s Hospital, Missouri Baptist Medical Center or affiliated Barnes Jewish Hospital testing sites located in Saint Louis, Missouri, USA. Inclusion criteria required that subjects were symptomatic and had a physician-ordered SARS-CoV-2 test performed in the course of their normal clinical care. Some subjects were enrolled before the return of the SARS-CoV-2 test result. Enrolled subjects who tested negative for SARS-CoV-2 are not included in the current manuscript. This report includes the first subjects enrolled in the study. The first 79 SARS-CoV-2^+^ subjects compose our primary cohort and the next 89 enrolled SARS-CoV-2^+^ subjects make up the validation cohort. Study recruitment is ongoing. All samples were collected at the time of enrollment, and there was a median interval between hospital admission and enrollment of 1 day (which corresponded to the median turn-around time for the SARS-CoV-2 clinical reverse transcription PCR test). Patient-reported duration of illness and other clinically relevant medical information was collected at the time of enrollment from the subject, their legally authorized representative, or the medical record. The vast majority of samples from patients testing positive for SARS-CoV-2 were collected immediately after the treatment team learned that the subjects were positive, and therefore, most patients will not have received any specific treatment before sample collected. We obtained less information about whether treatment had begun before sample collection for the validation cohort, hence, the consideration of the primary and validation cohorts separately throughout the majority of the manuscript. The portions of the study relevant to each institution were reviewed and approved by the Washington University in Saint Louis Institutional Review Board (WU-350 study approval no. 202003085) and the Missouri Baptist Medical Center Institutional Review Board (approval no. 1132). The study complied with the ethical standards of the Helsinki Declaration.

We also report findings from healthy control subjects and influenza-infected subjects enrolled in separate, ongoing studies. Control subjects had not experienced symptoms of a viral respiratory illness at the time of sample collection or within the previous 90 days, and samples were all collected before October of 2019. Influenza subjects were enrolled in the ongoing EDFLU study (*45*). All influenza subjects were sampled in 2019 and 2020. We enrolled most influenza subjects during the course of the 2019 to 2020 influenza season, immediately before the spread of COVID-19 disease in the Saint Louis region. The last included influenza subject was enrolled and sampled on 2 March 2020. The first case of COVID-19 was reported in Saint Louis on 8 March 2020 in a returning traveler. The control and influenza studies were independently approved by the Washington University Institutional Review Board (approval numbers 201707160, 201801209, 201808171, 201710220, 201808115, and 201910011).

### Multi-parameter flow cytometry

Absolute counts of CD45^+^ cells in whole blood were determined at the time of blood collection on fresh samples by flow cytometry with Precision Count Beads (BioLegend). PBMCs, prepared using ficoll separation, were analyzed using a panel of antibodies directed against the following antigens: CD8 BV421 (clone RPA-T8), CD20 Pacific Blue (clone 2H7), CD16 BV570 (clone 3G8), HLA-DR BV605 (clone L243), immunoglobulin D (IgD) SuperBright 702 (clone IA6-2), CD19 BV750 (clone HIB19), CD45 Alexa Fluor 532 (clone HI30), CD71 PE (clone CY1G4), CD38 PE-Cy7 (clone HIT2), CD14 APC (clone M5E2), CD4 Spark 685 (clone SK3), and CD3 Alexa 700 (clone UCHT1). PBMC samples of 0.5 to 2 × 10^6^ cells were stained with a master-mix containing pretitrated concentrations of the antibodies, along with BD Brilliant Buffer (BD Biosciences) and Zombie NIR Fixable Viability Marker (BioLegend) to differentiate live and dead cells. Samples were run on a Cytek Aurora spectral flow cytometer using SpectroFlo software (Cytek) and unmixed before final analysis was completed using FlowJo software (BD Biosciences).

### Cytokine quantification

Plasma obtained from subjects was frozen at −80°C and subsequently analyzed using a human magnetic cytokine panel providing parallel measurement of 35 cytokines (Thermo Fisher Scientific). The assay was performed according to the manufacturer’s instructions with each subject sample performed in duplicate and then analyzed on a Luminex FLEXMAP 3D instrument.

### Single-cell RNA sequencing

PBMCs were suspended at 1000 cells/μl, and approximately 17,400 cells were input to a 10× Genomics Chromium instrument. Aside from healthy control sample ZW-WU321, each sample was used for two independent reactions, with all first reactions processed on one chip and second reactions processed on a second chip. Single-cell gene expression libraries were prepared using 5′ (V2) kits and sequenced on the Illumina NovaSeq 6000 platform at 151 × 151 base pair. Individual libraries were processed using CellRanger (v3.1.0; 10× Genomics) with the accompanying human reference (GRCh38 3.0.0), which was modified to include the influenza A, influenza B, and COVID-19 (NC_045512.2) genomes. Processed libraries were subsequently aggregated using CellRanger, randomly subsampling mapped reads to equalize sequencing depth across cells. Filtered aggregation matrices were subsequently analyzed using Seurat (*46*) (v3.2.0), excluding cells from downstream analyses that exhibited extremes in the total number of transcripts expressed, the total number of genes expressed, or mitochondrial gene expression. For each cell, we inferred cell cycle phase using markers from Tirosh *et al.* (*47*) and incorporated module scores from a number of external gene sets in the same manner.

After filtering, we first sought to characterize putative cell subsets shared across conditions by detecting integration anchors among the samples, effectively minimizing condition-associated differences. The top 2000 variable genes were identified for each library using the “vst” method, and integration anchors were obtained using canonical correlation analysis (CCA). Data were integrated using 50 CCA dimensions and scaled to regress out the effects of total transcript count, percent of mitochondrial gene expression, and module scores associated with cell phase. Principal components (PC) were calculated and assessed for statistical significance using random permutation. The first 45 PCs (*P* < 0.01) were used to identify transcriptional clusters and for t-distributed stochastic neighbor embedding and UMAP (uniform manifold approximation and projection) dimensionality reduction. After identifying clusters on the basis of transcriptional similarities across cells from all three conditions (i.e., the “integrated” analysis), we performed pairwise differential gene expression analysis between conditions using Wilcoxon rank sum tests as implemented in Seurat, with default parameters. We also generated an additional UMAP projection using the top 2000 variable genes across the entire dataset (excluding T cell receptor and immunoglobulin (IG) genes, which are known to map poorly) irrespective of the CCA but again using significant PCs; this allowed us to visualize cells in a manner that did not obscure transcriptional differences owed to sample or condition but with previously identified cell subsets and transcriptional clusters from the integration analysis overlaid. We also looked within identified subsets and clusters for explicit differences in gene pathway enrichment between cells from COVID-19–infected and influenza-infected patients, COVID-19–infected and healthy participants, and influenza-infected and healthy participants. For these analyses, gene expression differences between conditions were ranked for individual subsets and transcriptional clusters by calculating differential expression under a generalized linear hurdle model (*48*). To generate gene ranks, gene-specific average log fold changes were multiplied by the absolute difference in the proportions of cells expressing the gene (1 × 10^−4^ as a lower boundary) and the inverse of the Bonferroni-corrected *P* values, which were rescaled from 1 × 10^−7^ to 1 to institute reasonable bounds in the ranking; this approach has been implemented previously (*49*) to synthesize information about average expression differences, the fraction of cells expressing the gene at all, and statistical assessments of significance. These ranks were used as inputs for gene set enrichment analysis (*26*) using GSEA Preranked with a classic enrichment statistic and chip-based gene collapsing on the basis of the Human_Symbol_with_Remapping_MSigDB.v.7.0 chip (*50*). Gene sets were considered significantly enriched if they resulted in a nominal *P* value of <0.05 and a *q* value of <0.20.

### Data availability

Raw flow cytometry and cytokine data can be found in table S1. Single-cell gene expression data have been uploaded to the National Center for Biotechnology Information (NCBI) Short Read Archive under BioProject ID PRJNA630932 and SRA accession SRR11233662.

### Analysis

#### Cohort comparisons

Statistical analyses of primary cohort demographics included pairwise comparisons between COVID-19 and healthy groups for age, sex, and ethnicity using a multinomial logistic regression, with the variation from the influenza group included in the model. Results were reported as odds ratio and (when significant) corresponding *P* value. Comparisons between COVID-19 and influenza groups were performed using a multivariate logistic regression between the COVID-19 and influenza groups only, as the clinical variables were irrelevant to healthy controls. The “immunocompromised” comorbidity was not included in the primary cohort logistic regression because it perfectly segregated across groups. The COVID-19 validation cohort was compared to the COVID-19, healthy, and influenza groups from the primary cohort again using multinomial logistic regression, with comparisons between validation and healthy groups limited to demographic variables. Multinomial regressions were modeled using the R “nnet” package (v7.3.14) (*51*), and multivariate logistic regressions were modeled using the “glm” function in R. *P* values were adjusted for multiple testing by controlling the Benjamini-Hochberg false discovery rate approach.

#### Flow cytometry

Flow cytometry measures were compared across healthy, influenza, and SARS-CoV-2 subjects using multivariate linear regression, with log_10_ subset percentages, counts, or mean fluorescence intensities modeled as a function of condition (COVID-19–infected, influenza-infected, and healthy control) with sex, age, ethnicity, and all comorbidities included as covariates among comparisons to healthy controls, and all of those covariates, as well as the number of days since symptom onset at study enrollment included in comparisons between influenza-infected and COVID-19–infected patients. The “emmeans” package in R was used to assess pairwise differences in estimated marginal means between conditions or severity, and Tukey’s method was used to adjust for multiple comparisons. In the HLA-DR expression analysis, there were four negative mean fluorescence intensity observations, and these were replaced with a value of 1 before analysis.

#### Cytokines

PCA was conducted on samples without missing data points using the “prcomp” function in R and visualized using the “factoextra” package. Cytokine concentrations were otherwise compared across healthy, influenza, and SARS-CoV-2 subjects using multivariate linear regression, with log_10_ concentration modeled as a function of condition (COVID-19–infected, influenza-infected, and healthy control) with sex, age, ethnicity, and all comorbidities included as covariates among comparisons to healthy controls, and all of those covariates, as well as the number of days since symptom onset at study enrollment, included in comparisons between influenza-infected and COVID-19–infected patients. The “emmeans” package in R was used to assess pairwise differences in estimated marginal means between conditions, and Tukey’s method was used to adjust for multiple comparisons. Data points from CSS samples were not included in the statistical analyses so as to prevent skewing the results. Data points from T_H_22 samples were included in the validation cohort COVID-19 samples for analysis even when visualized separately.

Cytokine-cytokine co-correlations were investigated using CytoMod (*24*) using absolute cytokine concentrations of COVID-19 samples across both the primary and validation cohorts. For these correlations, values below the lower limit of detection were set to the lower limit of detection, and values above the upper limit of detection were set to the upper limit of detection. We tested up to *k* = 12 modules and used the change in gap statistic to identify the optimal *k*.

Logistic regressions for severity were carried out within R and included sex, age, ethnicity, the number of days since symptom onset, cohort, and all comorbidities as covariates. *P* values were adjusted for multiple testing by controlling the false discovery rate as described above. Forest plots were generated using the “forestplot” package in R.

## Supplementary Material

http://advances.sciencemag.org/cgi/content/full/sciadv.abe3024/DC1

Table S1

Table S2

Adobe PDF - abe3024_SM.pdf

Distinct inflammatory profiles distinguish COVID-19 from influenza with limited contributions from cytokine storm

## SUPPLEMENTARY MATERIALS

Supplementary material for this article is available at http://advances.sciencemag.org/cgi/content/full/sciadv.abe3024/DC1

## Appendix

View/request a protocol for this paper from *Bio-protocol*.
