# Supplementary material for: Distinct inflammatory profiles distinguish COVID-19 from influenza with limited contributions from cytokine storm
Source: Sci Adv. 2020 Dec 9;6(50):eabe3024. doi: 10.1126/sciadv.abe3024 (PMC7725462; doi:10.1126/sciadv.abe3024)
Supplement: http://advances.sciencemag.org/cgi/content/full/sciadv.abe3024/DC1 [file abe3024_index.html]

Science Advances | Science AdvancesAAASSearchScience AdvancesMenu

## Supplementary Materials

# Distinct inflammatory profiles distinguish COVID-19 from influenza with limited contributions from cytokine storm

Philip A. Mudd, Jeremy Chase Crawford, Jackson S. Turner, Aisha Souquette, Daniel Reynolds, Diane Bender, James P. Bosanquet, Nitin J. Anand, David A. Striker, R. Scott Martin, Adrianus C. M. Boon, Stacey L. House, Kenneth E. Remy, Richard S. Hotchkiss, Rachel M. Presti, Jane A. O’Halloran, William G. Powderly, Paul G. Thomas and Ali H. Ellebedy

Download Supplement

**The PDF file includes:**

- Figs. S1 to S13

**Other Supplementary Material for this manuscript includes the following:**

- Table S1
- Table S2

**Files in this Data Supplement:**

- Adobe PDF - abe3024\_SM.pdf
- abe3024\_Table\_S1.xlsx
- abe3024\_Table\_S2.rar
