# Supplementary material for: Distinct inflammatory profiles distinguish COVID-19 from influenza with limited contributions from cytokine storm
Source: Sci Adv. 2020 Dec 9;6(50):eabe3024. doi: 10.1126/sciadv.abe3024 (PMC7725462; doi:10.1126/sciadv.abe3024)
Supplement: http://advances.sciencemag.org/cgi/content/full/sciadv.abe3024/DC1 [file abe3024_1.pdf]

Science Advances
AAAS

advances.sciencemag.org/cgi/content/full/sciadv.abe3024/DC1

Supplementary Materials for

Distinct inflammatory profiles distinguish COVID-19 from influenza with limited contributions from cytokine storm

Philip A. Mudd*, Jeremy Chase Crawford, Jackson S. Turner, Aisha Souquette, Daniel Reynolds, Diane Bender, James P. Bosanquet, Nitin J. Anand, David A. Striker, R. Scott Martin, Adrianus C. M. Boon, Stacey L. House, Kenneth E. Remy, Richard S. Hotchkiss, Rachel M. Presti, Jane A. O'Halloran, William G. Powderly, Paul G. Thomas* and Ali H. Ellebedy*

*Corresponding author. Email: pmudd@wustl.edu, paul.thomas@stjude.org, and ellebedy@wustl.edu

Published 13 November 2020, *Sci. Adv.* **6**, eabe3024 (2020)
DOI: 10.1126/sciadv.abe3024

This PDF file includes:

Figs. S1 to S13

Other Supplementary Material for this manuscript includes the following:

(available at advances.sciencemag.org/cgi/content/full/sciadv.abe3024/DC1)

Tables S1 to S2

Supplementary Tables are available for download separately.

Supplementary Table 1. Flow cytometry, cytokine, and metadata for all study subjects. In the "Data" tab, columns A-Y contain metadata (e.g., age, ethnicity), Z-BH are cytokines levels (in pg/mL), and BI-CI contain flow cytometry results. Descriptions of metadata variables are located in the "Dictionary" tab.

Supplementary Table 2. COVID-19 versus influenza differential gene expression results by major cell subsets. Differential gene expression analysis was conducted in Seurat, comparing cells from COVID-19-infected patients to those from influenza-infected patients for each major subset annotated in clustering analyses. A positive average log fold change (avg logFC) indicates an upregulation among COVID-19 relative to influenza. Pct.1 corresponds to the percentage of cells from COVID-19 subjects within a given subset expressing a given gene, whereas Pct.2 corresponds to the percentage of cells from influenza subjects within a given subset expressing a given gene. The statistical test utilized was "MAST", with no log fold change or minimum percent threshold. P-values were adjusted for multiple comparisons using Bonferroni correction.

Supplementary Figure 1:

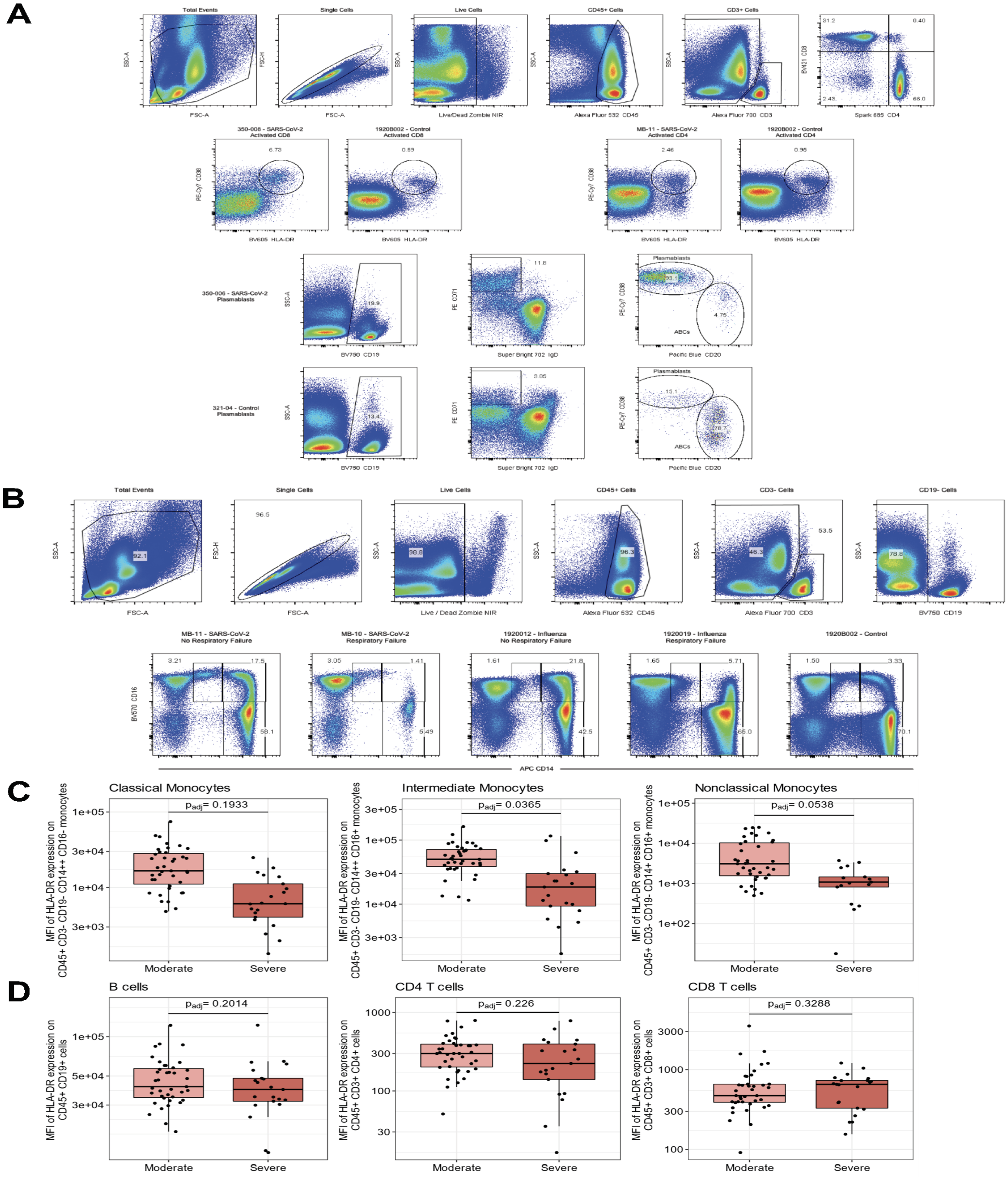

Supplementary Figure 1. Gating strategy for flow cytometry analyses. Gating strategy and representative plots for lymphocyte **(A)** and monocyte **(B)** subsets. Evaluation of HLA-DR abundance as a function of disease severity on monocyte **(C)** and lymphocyte **(D)** subpopulations in select SARS-CoV-2-infected subjects (N=22) as measured by geometric mean fluorescence intensity (MFI) using flow cytometry. Severe patients were those who required intubation and mechanical ventilation or who expired as a result of their illness, whereas moderately ill patients were all others who sought hospital care for their illness. Presented p-values are from pairwise comparisons of estimated marginal means of linear regression models that adjust for ethnicity, sex, age, days of symptom duration at study enrollment, and all comorbidities (immunocompromised, end stage renal disease, chronic lung disease, chronic heart failure, and diabetes mellitus). P-values were adjusted for multiple comparisons using Tukey’s method.

Supplemental Figure 2.

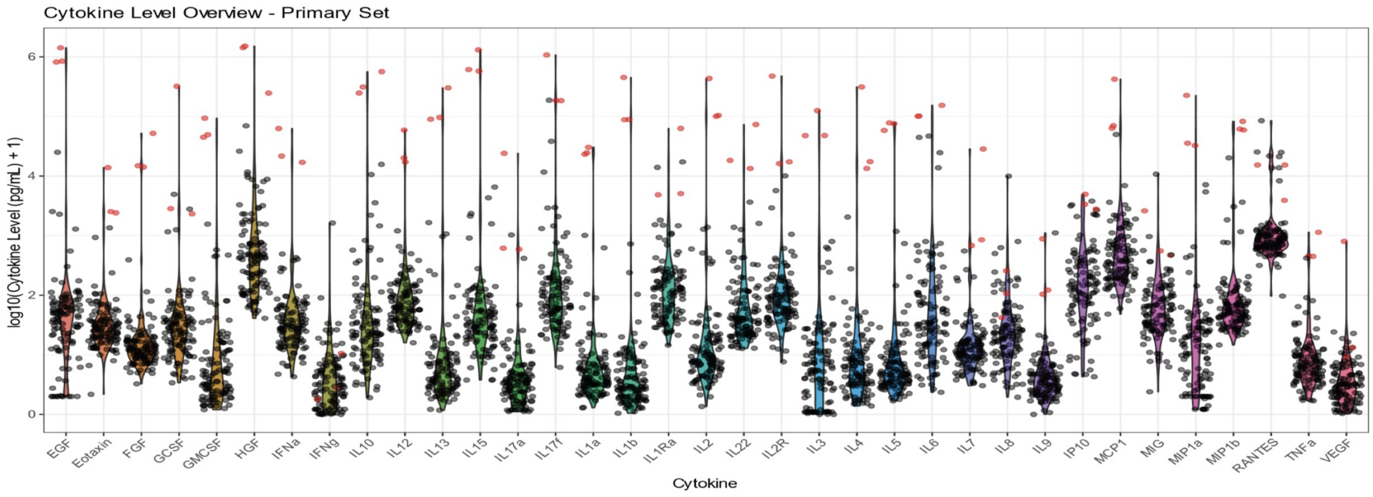

Supplementary Figure 2. Overview of cytokine levels across all primary cohort subjects. Data points from the 3 COVID-19 patients with extremely high cytokine concentrations (deemed cytokine storm profiles) are shown in red.

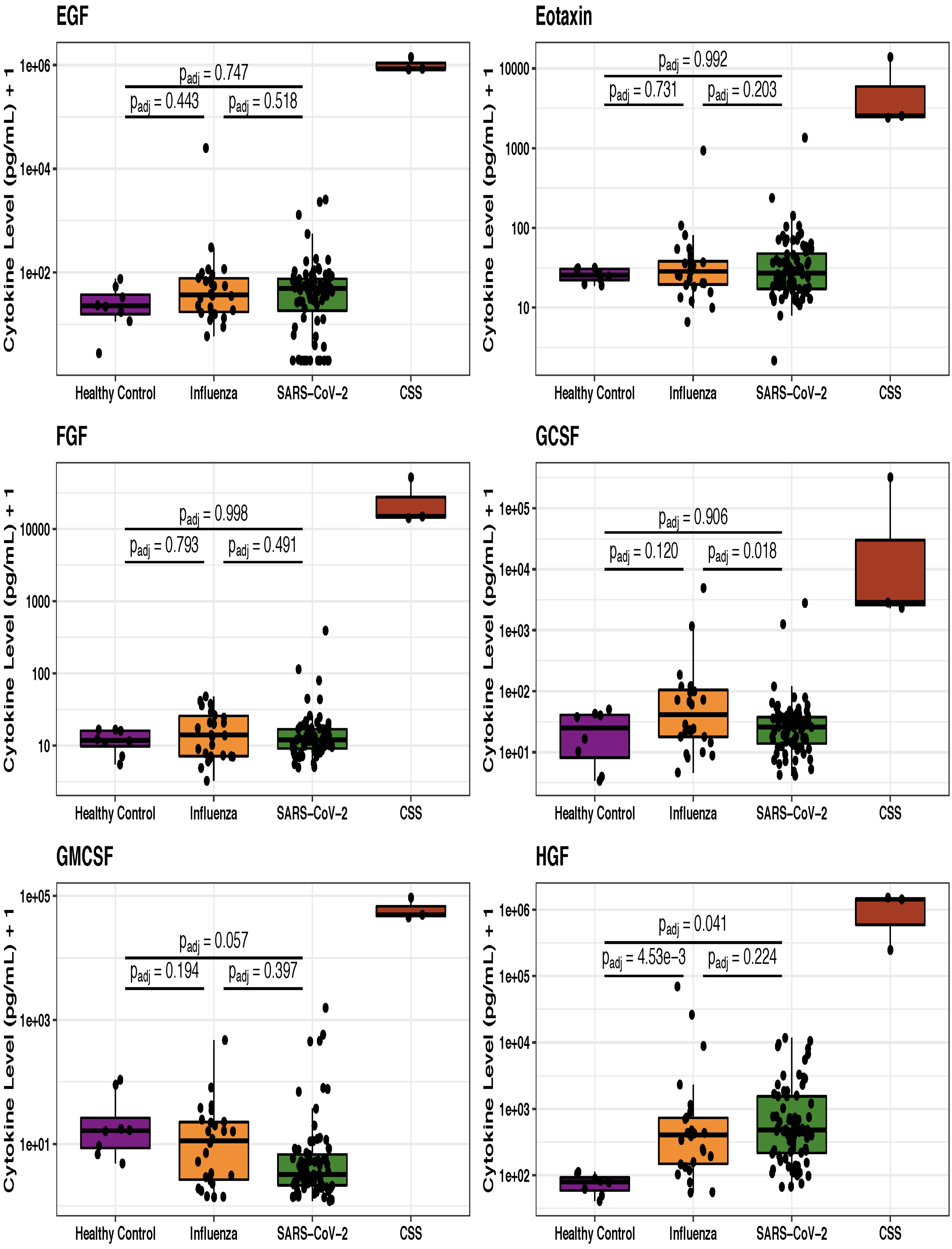

EGF
Cytokine Level (pg/mL) + 1
1e+06
1e+04
1e+02
p_adj = 0.747
p_adj = 0.443
p_adj = 0.518
Healthy Control
Influenza
SARS-CoV-2
CSS
Eotaxin
Cytokine Level (pg/mL) + 1
10000
1000
100
10
p_adj = 0.992
p_adj = 0.731
p_adj = 0.203
Healthy Control
Influenza
SARS-CoV-2
CSS
FGF
Cytokine Level (pg/mL) + 1
10000
1000
100
10
p_adj = 0.998
p_adj = 0.793
p_adj = 0.491
Healthy Control
Influenza
SARS-CoV-2
CSS
GCSF
Cytokine Level (pg/mL) + 1
1e+05
1e+04
1e+03
1e+02
1e+01
p_adj = 0.906
p_adj = 0.120
p_adj = 0.018
Healthy Control
Influenza
SARS-CoV-2
CSS
GMCSF
Cytokine Level (pg/mL) + 1
1e+05
1e+03
1e+01
p_adj = 0.057
p_adj = 0.194
p_adj = 0.397
Healthy Control
Influenza
SARS-CoV-2
CSS
HGF
Cytokine Level (pg/mL) + 1
1e+06
1e+05
1e+04
1e+03
1e+02
p_adj = 0.041
p_adj = 4.53e-3
p_adj = 0.224
Healthy Control
Influenza
SARS-CoV-2
CSS

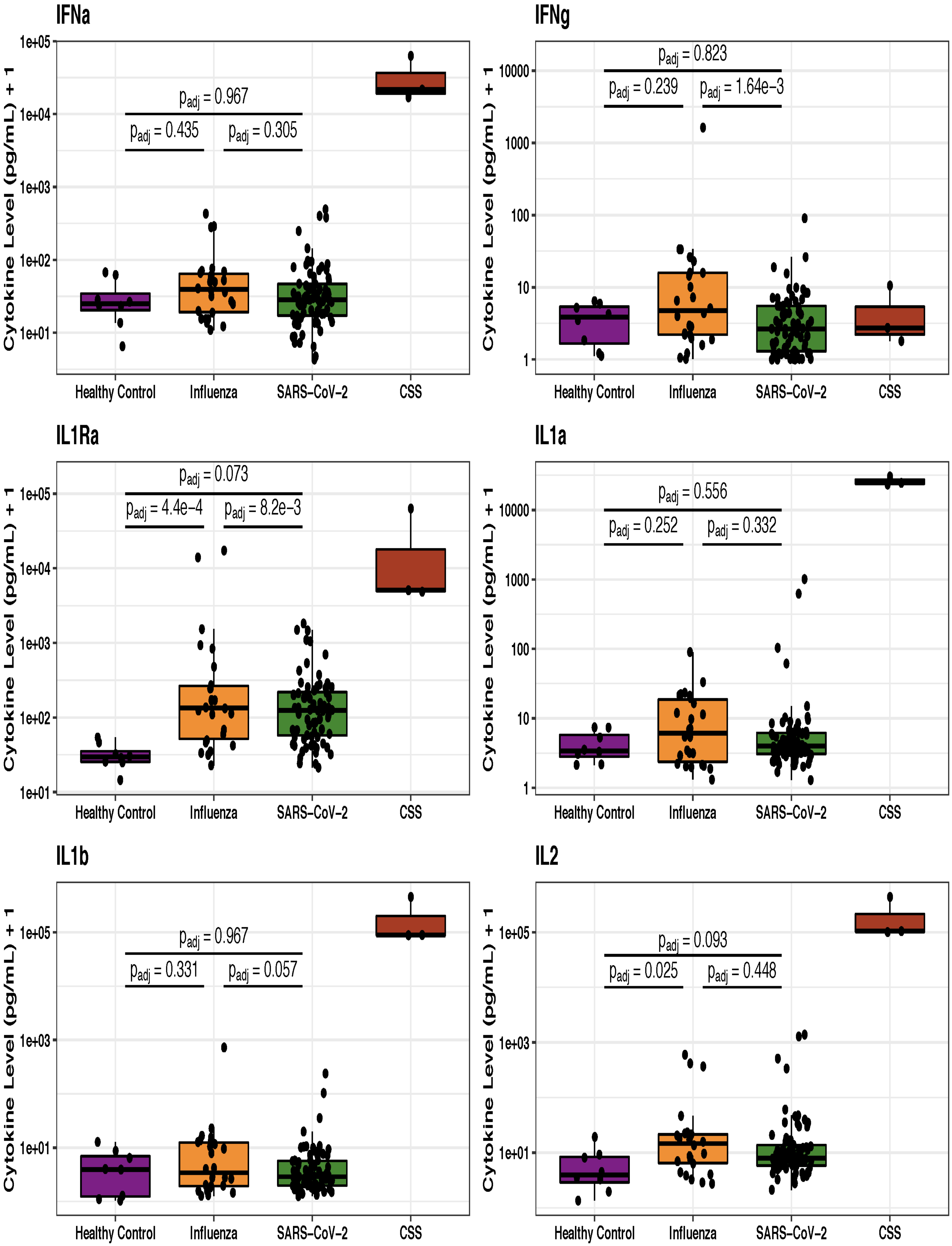
IFNa
padj = 0.967
padj = 0.435
padj = 0.305
1e+05
1e+04
1e+03
1e+02
1e+01
Cytokine Level (pg/mL) + 1
Healthy Control
Influenza
SARS−CoV−2
CSS
IFNg
padj = 0.823
padj = 0.239
padj = 1.64e−3
10000
1000
100
10
1
Cytokine Level (pg/mL) + 1
Healthy Control
Influenza
SARS−CoV−2
CSS
IL1Ra
padj = 0.073
padj = 4.4e−4
padj = 8.2e−3
1e+05
1e+04
1e+03
1e+02
1e+01
Cytokine Level (pg/mL) + 1
Healthy Control
Influenza
SARS−CoV−2
CSS
IL1a
padj = 0.556
padj = 0.252
padj = 0.332
10000
1000
100
10
1
Cytokine Level (pg/mL) + 1
Healthy Control
Influenza
SARS−CoV−2
CSS
IL1b
padj = 0.967
padj = 0.331
padj = 0.057
1e+05
1e+03
1e+01
Cytokine Level (pg/mL) + 1
Healthy Control
Influenza
SARS−CoV−2
CSS
IL2
padj = 0.093
padj = 0.025
padj = 0.448
1e+05
1e+03
1e+01
Cytokine Level (pg/mL) + 1
Healthy Control
Influenza
SARS−CoV−2
CSS

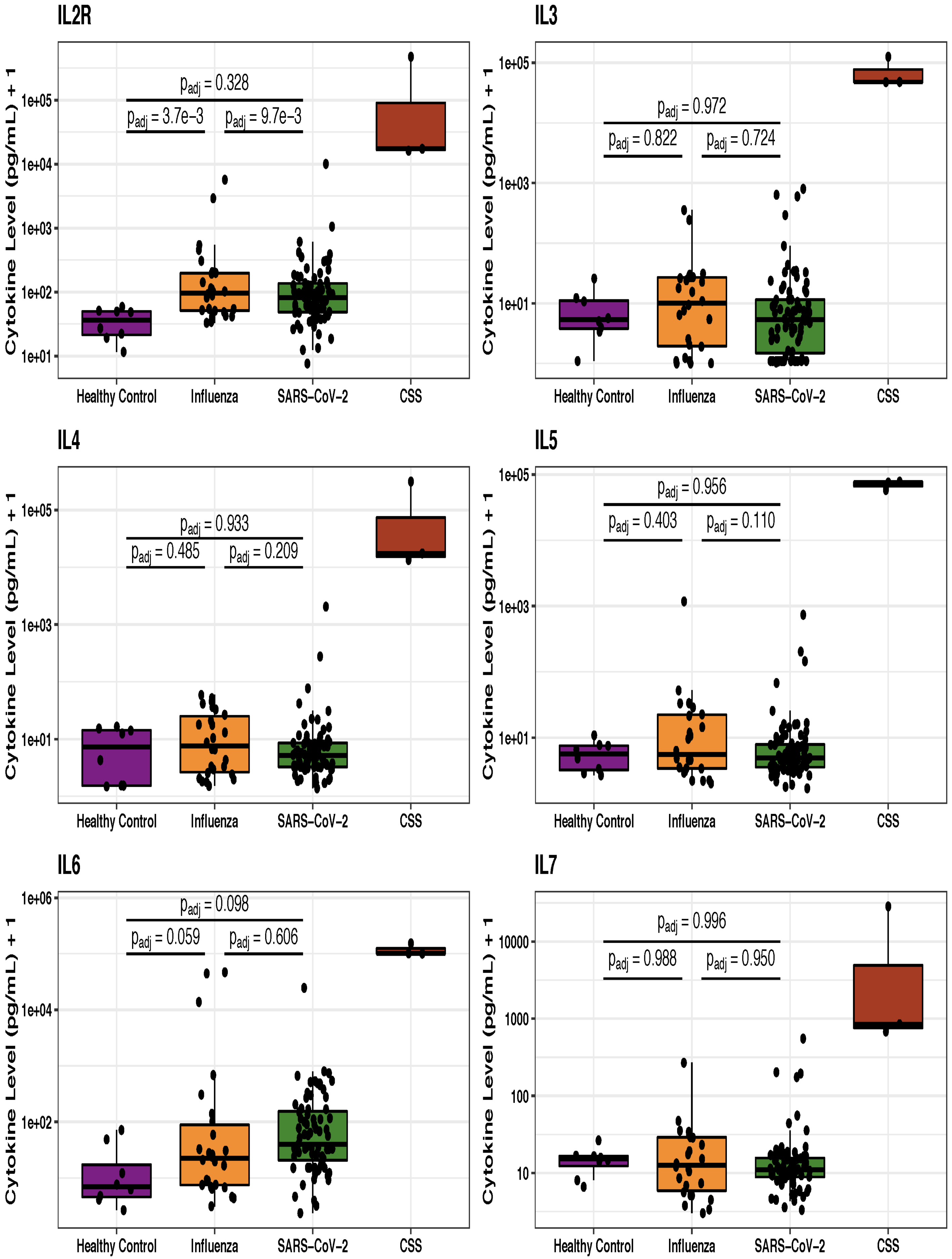

IL2R
Cytokine Level (pg/mL) + 1
1e+01
1e+02
1e+03
1e+04
1e+05
p_{adj} = 0.328
p_{adj} = 3.7e−3
p_{adj} = 9.7e−3
Healthy Control
Influenza
SARS−CoV−2
CSS
IL3
Cytokine Level (pg/mL) + 1
1e+01
1e+03
1e+05
p_{adj} = 0.972
p_{adj} = 0.822
p_{adj} = 0.724
Healthy Control
Influenza
SARS−CoV−2
CSS
IL4
Cytokine Level (pg/mL) + 1
1e+01
1e+03
1e+05
p_{adj} = 0.933
p_{adj} = 0.485
p_{adj} = 0.209
Healthy Control
Influenza
SARS−CoV−2
CSS
IL5
Cytokine Level (pg/mL) + 1
1e+01
1e+03
1e+05
p_{adj} = 0.956
p_{adj} = 0.403
p_{adj} = 0.110
Healthy Control
Influenza
SARS−CoV−2
CSS
IL6
Cytokine Level (pg/mL) + 1
1e+02
1e+04
1e+06
p_{adj} = 0.098
p_{adj} = 0.059
p_{adj} = 0.606
Healthy Control
Influenza
SARS−CoV−2
CSS
IL7
Cytokine Level (pg/mL) + 1
10
100
1000
10000
p_{adj} = 0.996
p_{adj} = 0.988
p_{adj} = 0.950
Healthy Control
Influenza
SARS−CoV−2
CSS

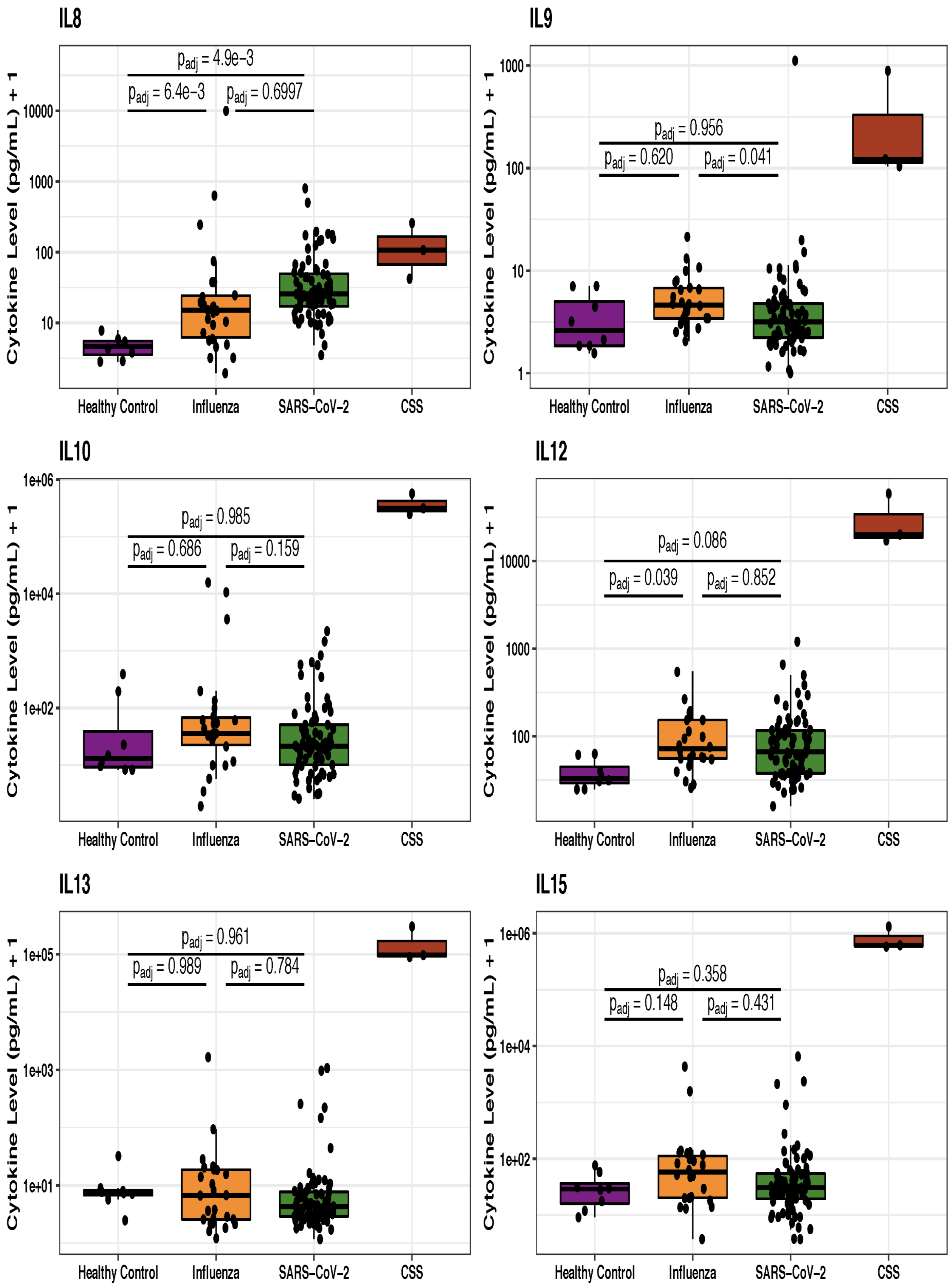

IL8
padj = 4.9e−3
padj = 6.4e−3
padj = 0.6997
IL9
padj = 0.956
padj = 0.620
padj = 0.041
IL10
padj = 0.985
padj = 0.686
padj = 0.159
IL12
padj = 0.086
padj = 0.039
padj = 0.852
IL13
padj = 0.961
padj = 0.989
padj = 0.784
IL15
padj = 0.358
padj = 0.148
padj = 0.431
Cytokine Level (pg/mL) + 1
Healthy Control
Influenza
SARS−CoV−2
CSS

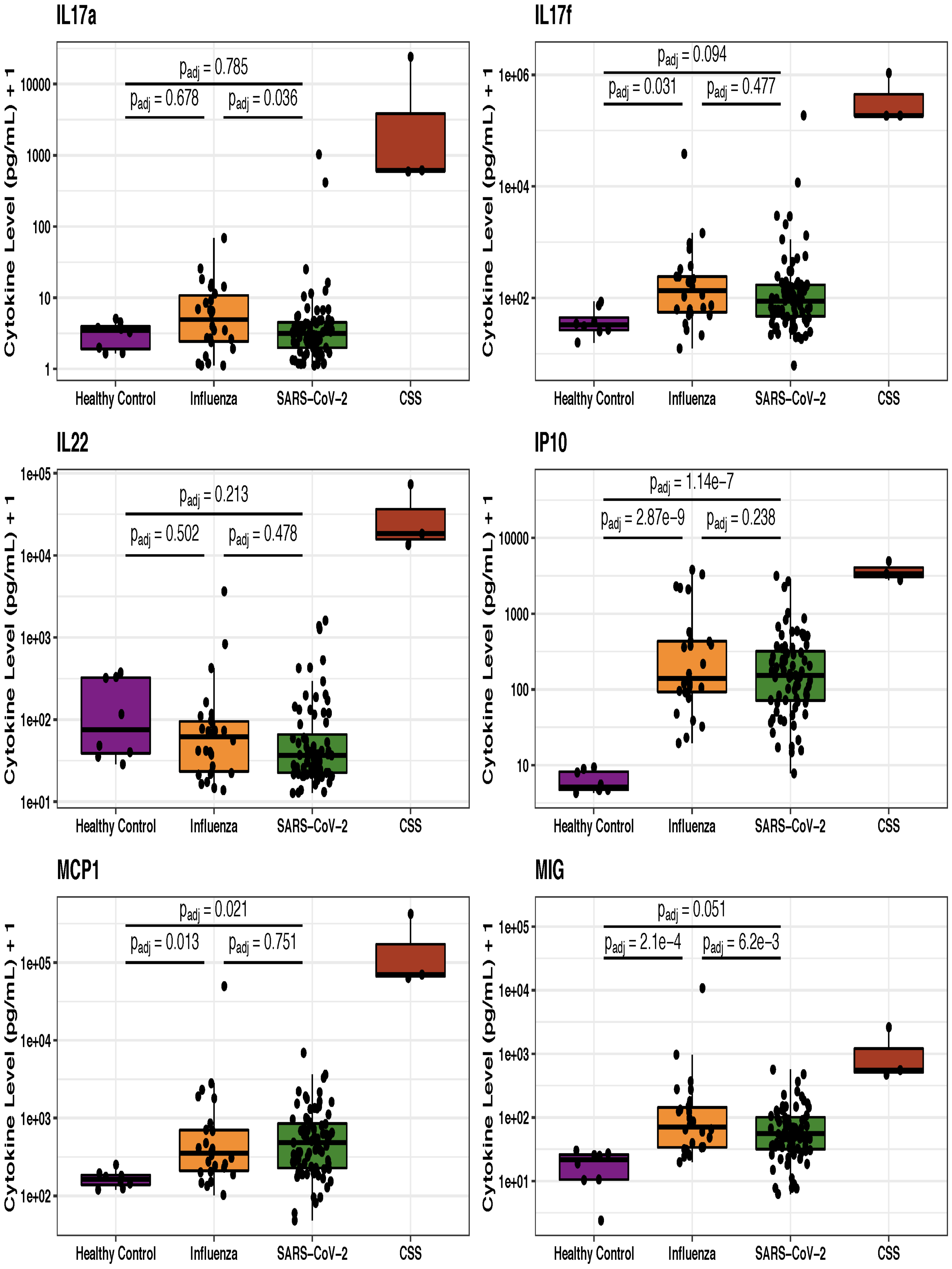
IL17a
p_adj = 0.785
p_adj = 0.678
p_adj = 0.036
IL17f
p_adj = 0.094
p_adj = 0.031
p_adj = 0.477
IL22
p_adj = 0.213
p_adj = 0.502
p_adj = 0.478
IP10
p_adj = 1.14e−7
p_adj = 2.87e−9
p_adj = 0.238
MCP1
p_adj = 0.021
p_adj = 0.013
p_adj = 0.751
MIG
p_adj = 0.051
p_adj = 2.1e−4
p_adj = 6.2e−3
Cytokine Level (pg/mL) + 1
Healthy Control
Influenza
SARS−CoV−2
CSS

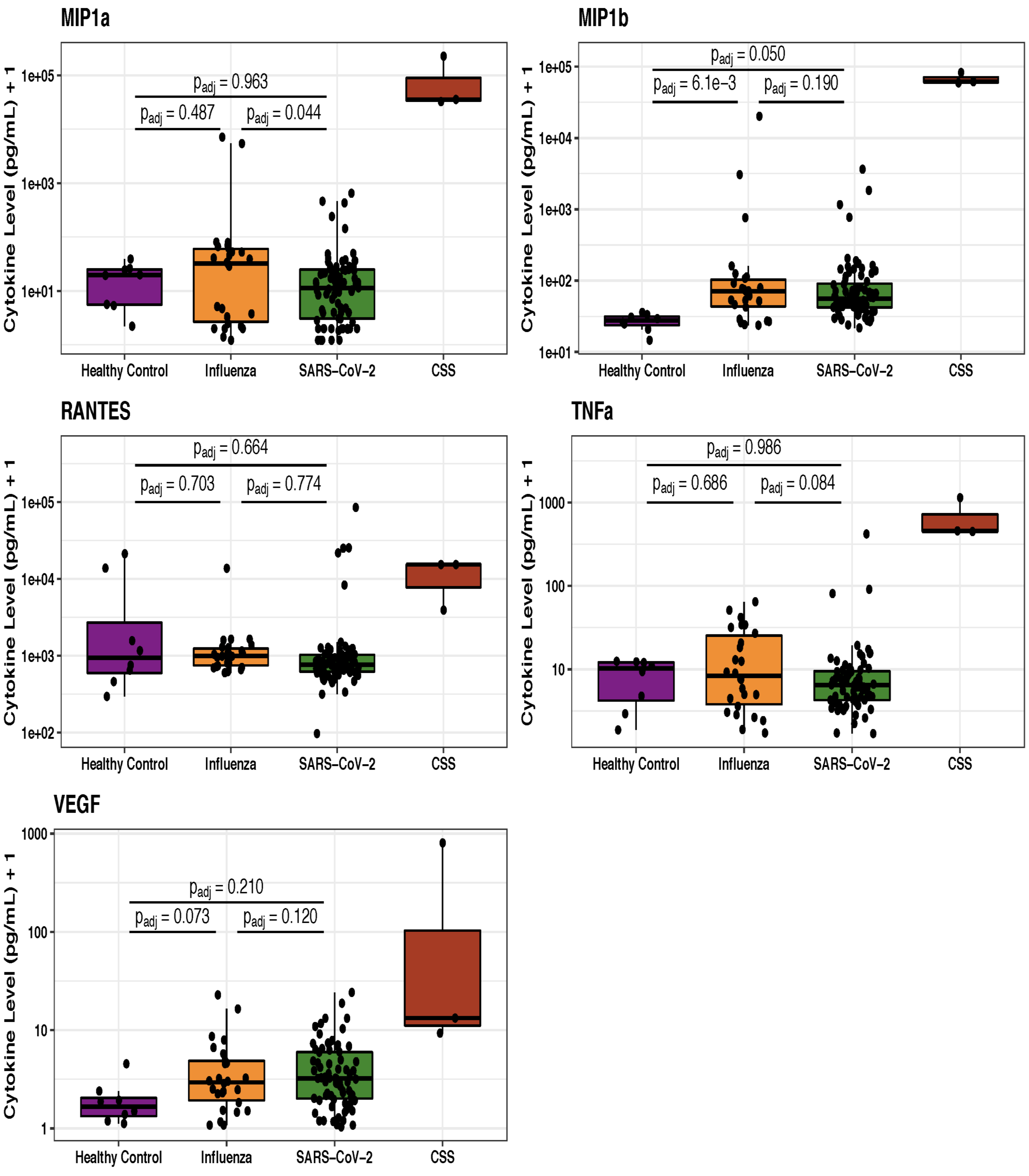

Supplementary Figure 3. Box plots show cytokine concentrations in healthy, influenza, COVID-19, and CSS (cytokine storm syndrome) subjects for all 35 cytokines measured, with raw values plotted on the log10 scale. Presented p-values are from estimated marginal means (EMM) comparisons, averaging over all demographic and clinical factors that were included as covariates and p-values adjusted for multiple comparisons.

Supplementary Figure 4: Primary Flu-Covid EMM Plots

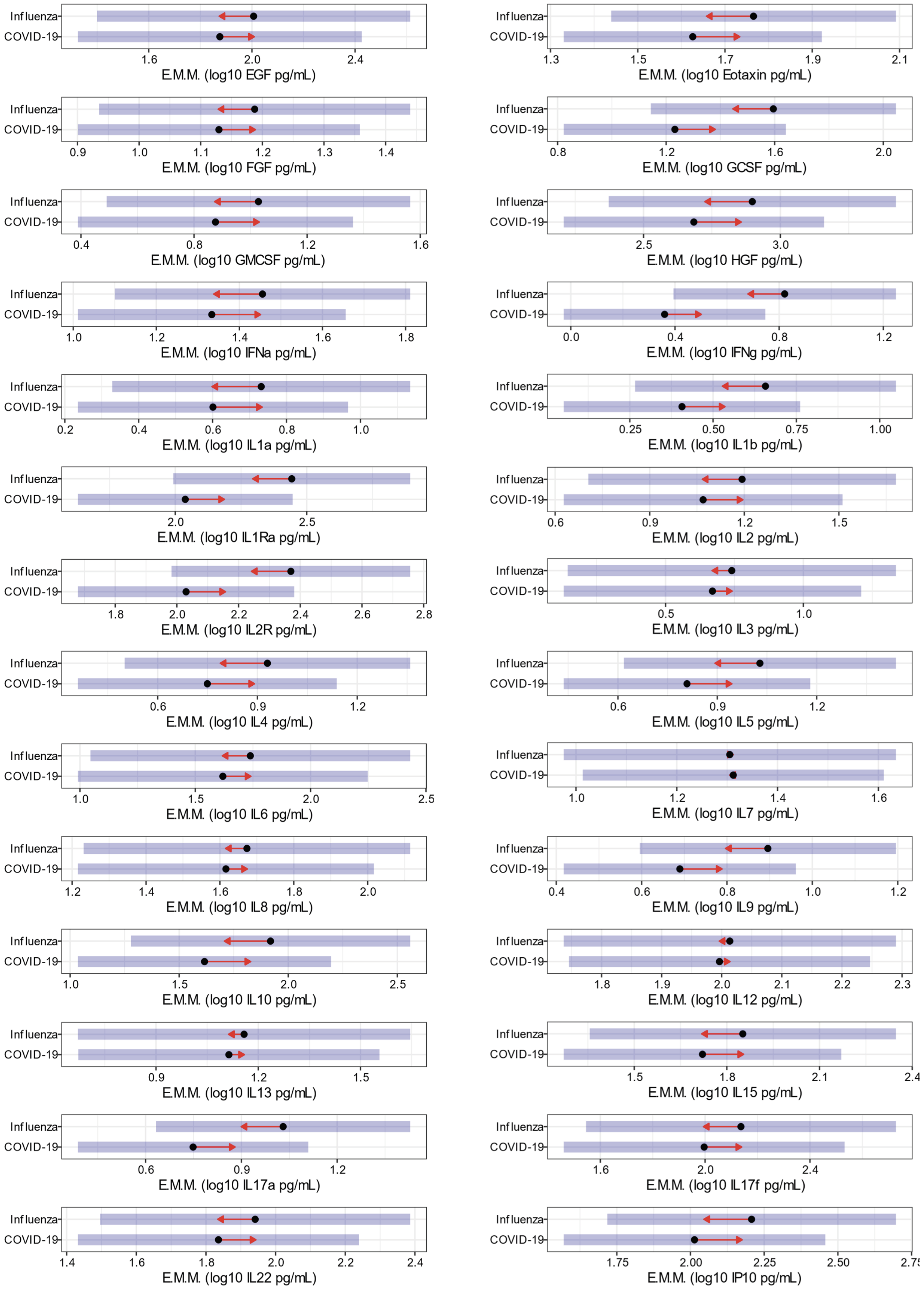

Supplementary Figure 4 part 2

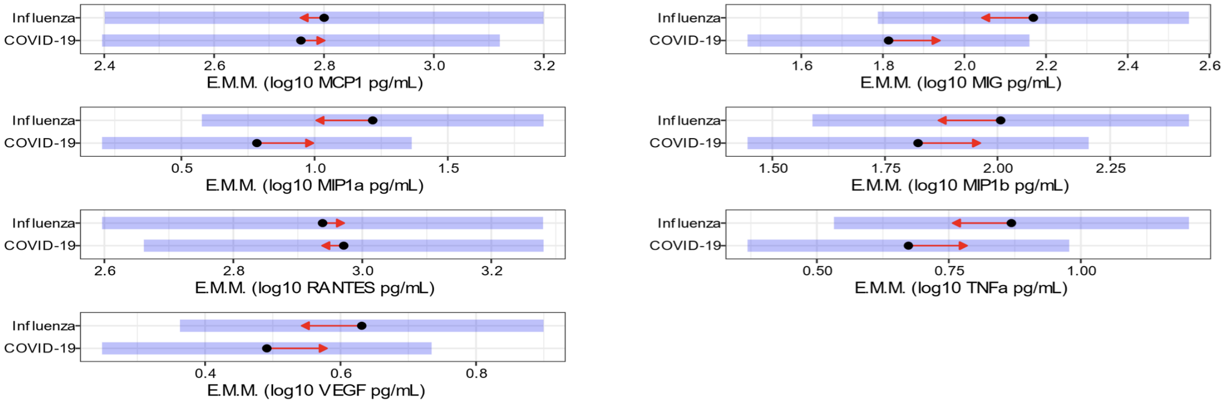

Supplementary Figure 4. Estimated marginal means (EMM) plots comparing cytokine expression between influenza and primary COVID-19 patients. The black dot represents the estimated marginal mean for the log10 concentration of the cytokine for a given condition, averaged over the levels of all other covariates (e.g. age, sex, ethnicity), and the blue shading represents the corresponding 95% confidence interval. The red arrows represent the standard error (SE) in one direction. Overlapping SE arrows indicate no significant difference between the EMM of a given cytokine in influenza subjects versus COVID-19 subjects.

Supplemental Figure 5.

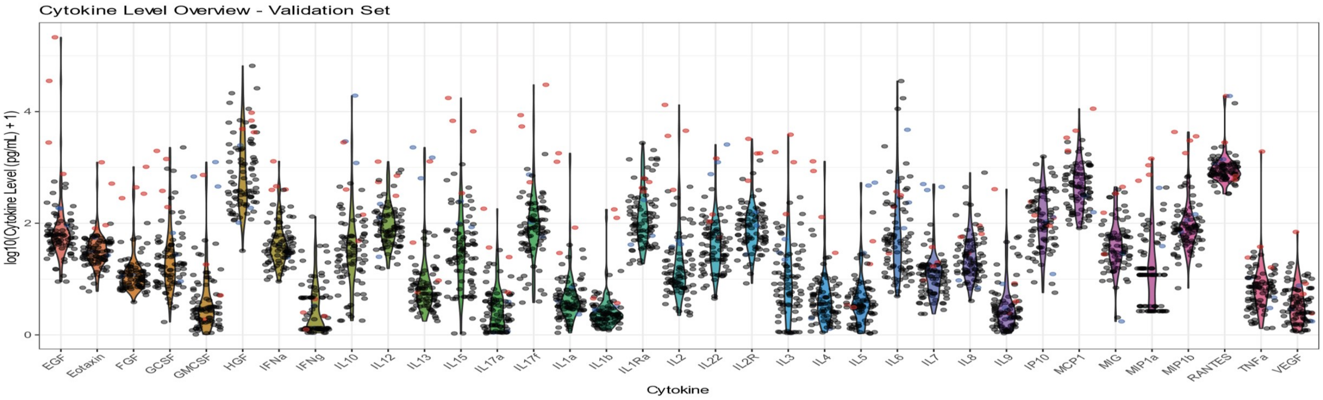

Supplementary Figure 5. Overview of cytokine levels across all validation cohort subjects (all of which were positive for COVID-19). Data points from the 4 patients with CSS (cytokine storm syndrome) profiles are shown in red, and data points from the 3 patients with Th22 cytokine profiles are shown in blue.

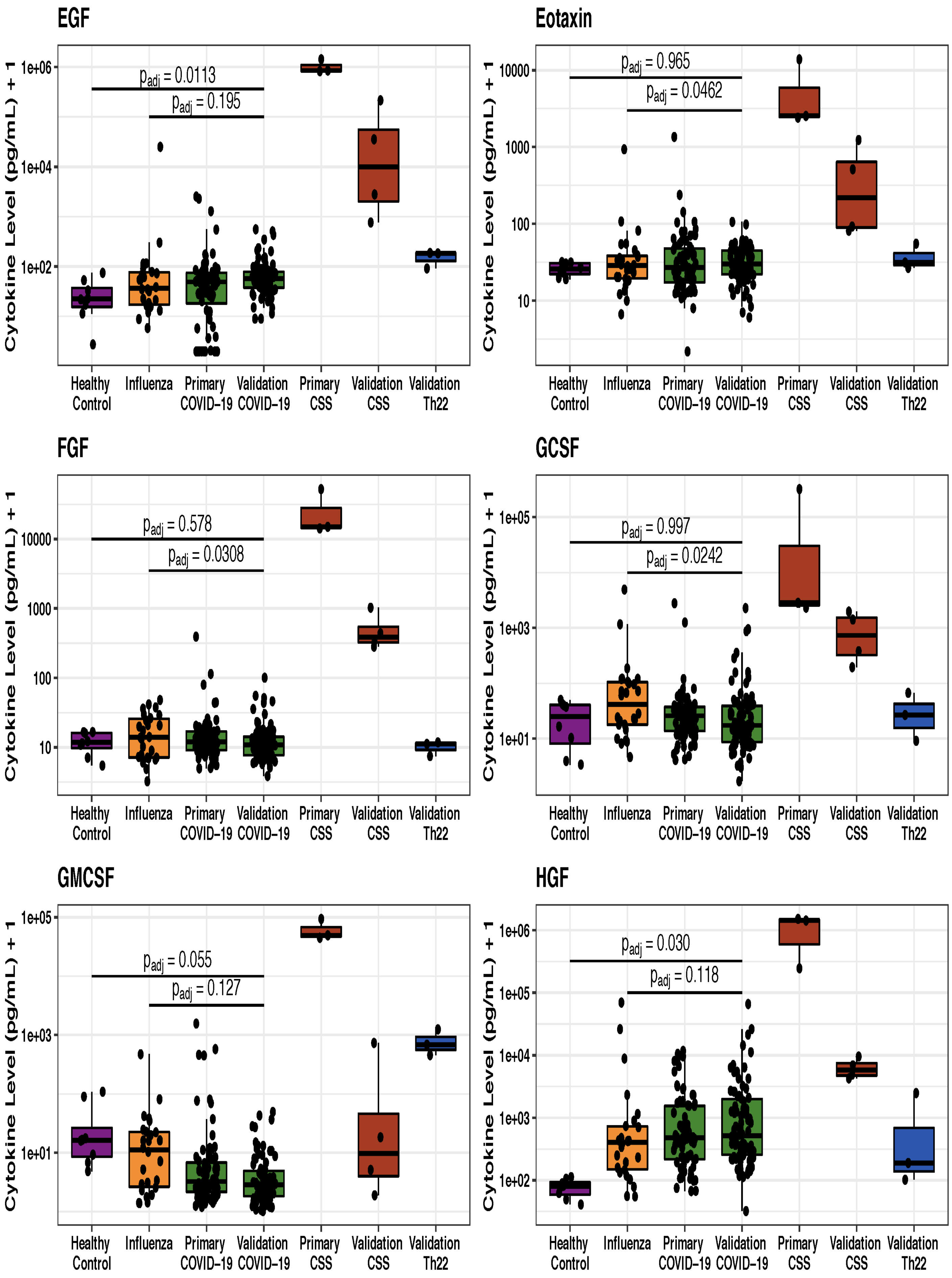
EGF
p_{adj} = 0.0113
p_{adj} = 0.195
Eotaxin
p_{adj} = 0.965
p_{adj} = 0.0462
FGF
p_{adj} = 0.578
p_{adj} = 0.0308
GCSF
p_{adj} = 0.997
p_{adj} = 0.0242
GMCSF
p_{adj} = 0.055
p_{adj} = 0.127
HGF
p_{adj} = 0.030
p_{adj} = 0.118
Cytokine Level (pg/mL) + 1
1e+01
1e+02
1e+03
1e+04
1e+05
1e+06
10
100
1000
10000
Healthy Control
Influenza
Primary COVID−19
Validation COVID−19
Primary CSS
Validation CSS
Validation Th22

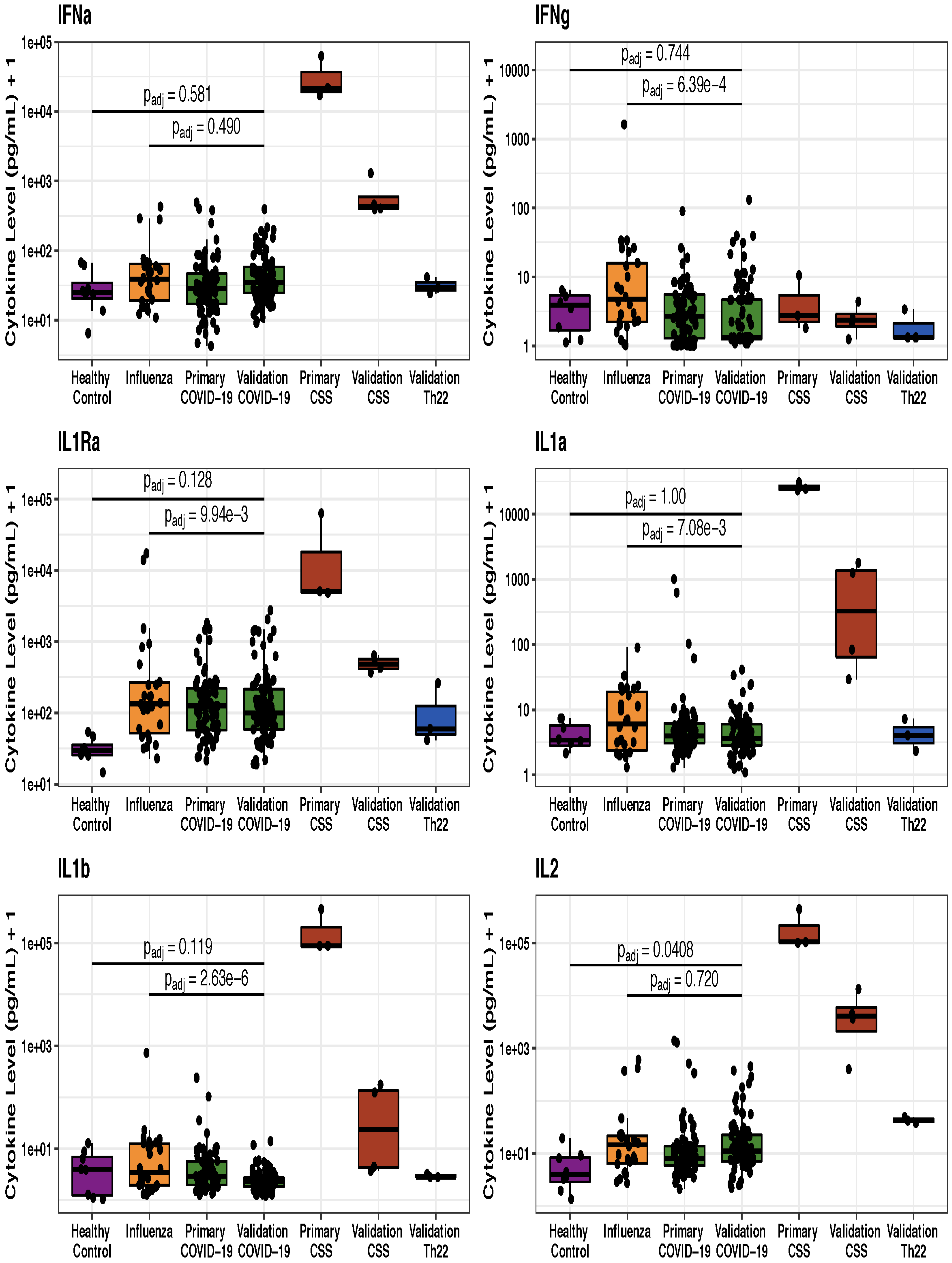
IFNa
IFNg
IL1Ra
IL1a
IL1b
IL2
Cytokine Level (pg/mL) + 1
p_adj = 0.581
p_adj = 0.490
p_adj = 0.744
p_adj = 6.39e−4
p_adj = 0.128
p_adj = 9.94e−3
p_adj = 1.00
p_adj = 7.08e−3
p_adj = 0.119
p_adj = 2.63e−6
p_adj = 0.0408
p_adj = 0.720
1e+01
1e+02
1e+03
1e+04
1e+05
1
10
100
1000
10000
Healthy Control
Influenza
Primary COVID−19
Validation COVID−19
Primary CSS
Validation CSS
Validation Th22

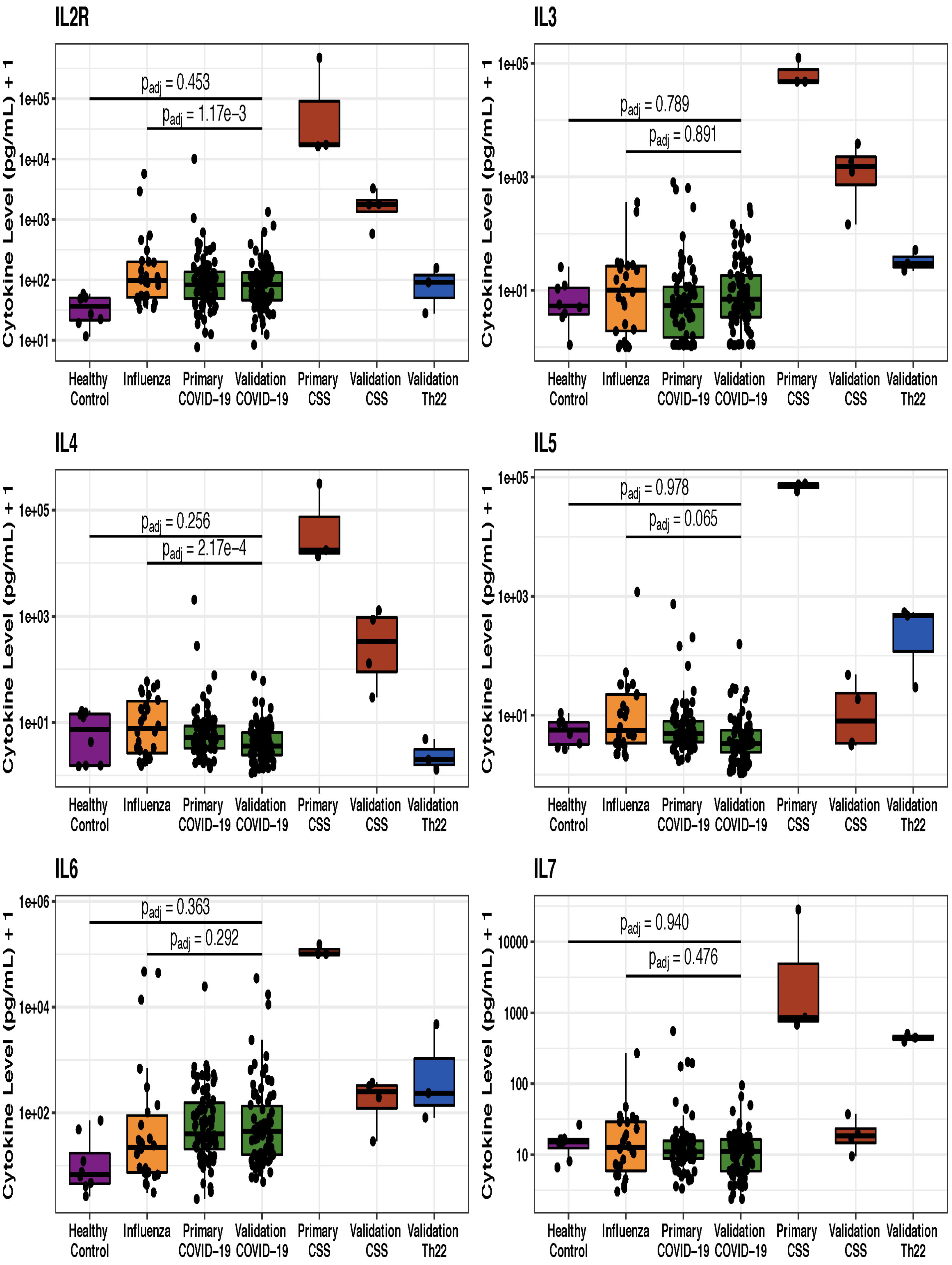
IL2R
p_{adj} = 0.453
p_{adj} = 1.17e−3
IL3
p_{adj} = 0.789
p_{adj} = 0.891
IL4
p_{adj} = 0.256
p_{adj} = 2.17e−4
IL5
p_{adj} = 0.978
p_{adj} = 0.065
IL6
p_{adj} = 0.363
p_{adj} = 0.292
IL7
p_{adj} = 0.940
p_{adj} = 0.476
Cytokine Level (pg/mL) + 1
1e+01
1e+02
1e+03
1e+04
1e+05
1e+06
10
100
1000
10000
Healthy Control
Influenza
Primary COVID−19
Validation COVID−19
Primary CSS
Validation CSS
Validation Th22

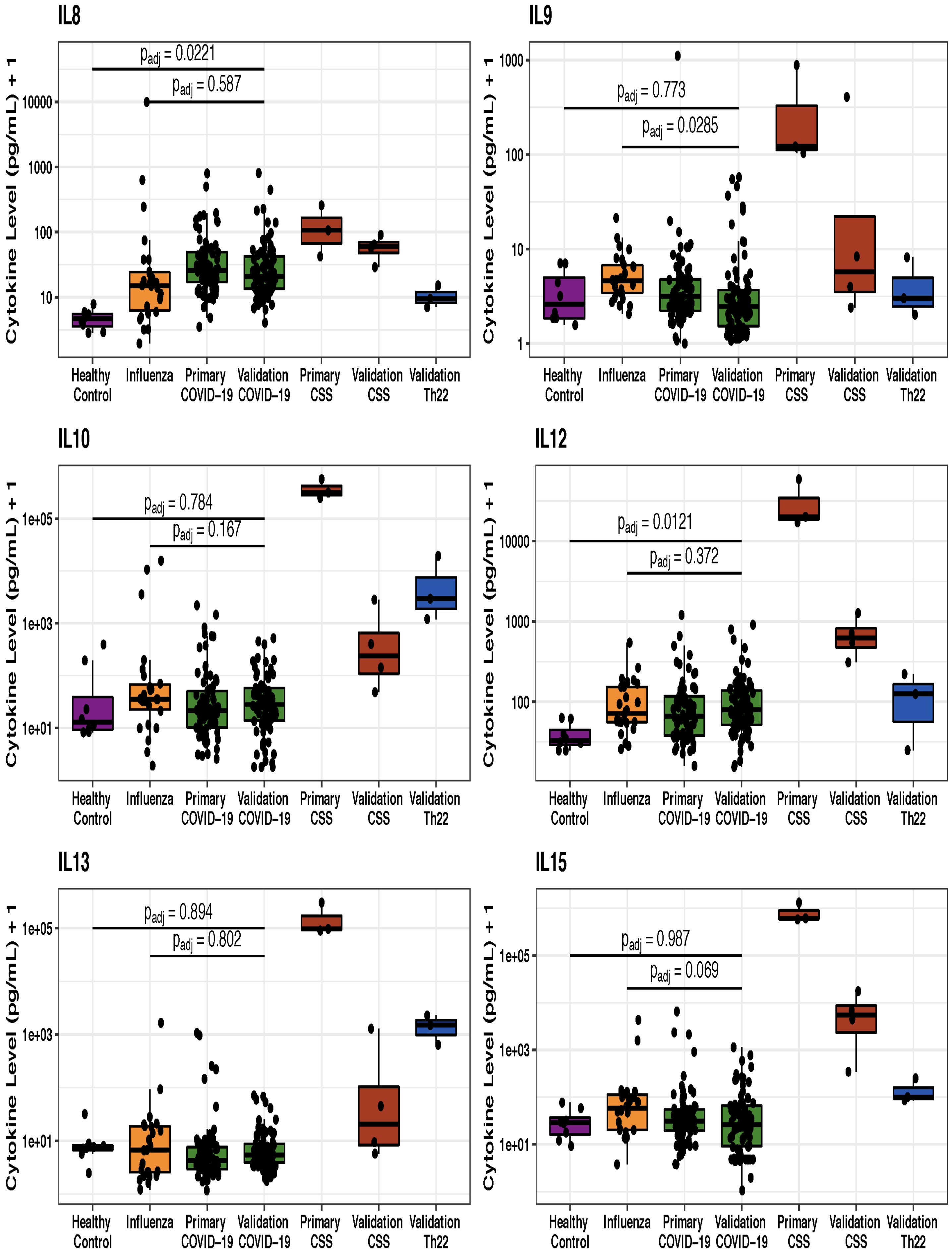
IL8
IL9
IL10
IL12
IL13
IL15
Cytokine Level (pg/mL) + 1
p_{adj} = 0.0221
p_{adj} = 0.587
p_{adj} = 0.773
p_{adj} = 0.0285
p_{adj} = 0.784
p_{adj} = 0.167
p_{adj} = 0.0121
p_{adj} = 0.372
p_{adj} = 0.894
p_{adj} = 0.802
p_{adj} = 0.987
p_{adj} = 0.069
10
100
1000
10000
1
1e+01
1e+03
1e+05
Healthy Control
Influenza
Primary COVID−19
Validation COVID−19
Primary CSS
Validation CSS
Validation Th22

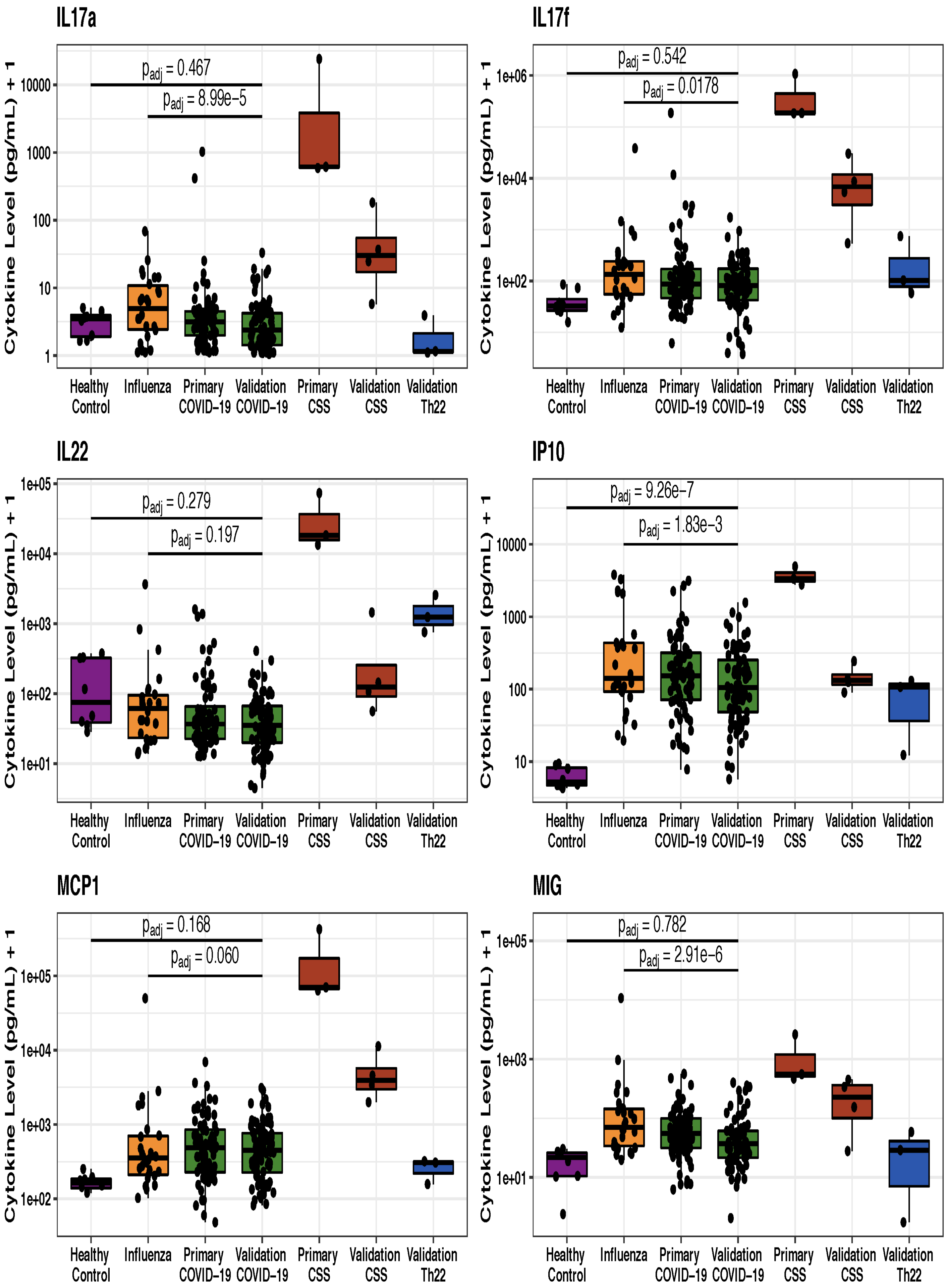
IL17a
p_{adj} = 0.467
p_{adj} = 8.99e−5
IL17f
p_{adj} = 0.542
p_{adj} = 0.0178
IL22
p_{adj} = 0.279
p_{adj} = 0.197
IP10
p_{adj} = 9.26e−7
p_{adj} = 1.83e−3
MCP1
p_{adj} = 0.168
p_{adj} = 0.060
MIG
p_{adj} = 0.782
p_{adj} = 2.91e−6
Cytokine Level (pg/mL) + 1
Healthy Control
Influenza
Primary COVID−19
Validation COVID−19
Primary CSS
Validation CSS
Validation Th22

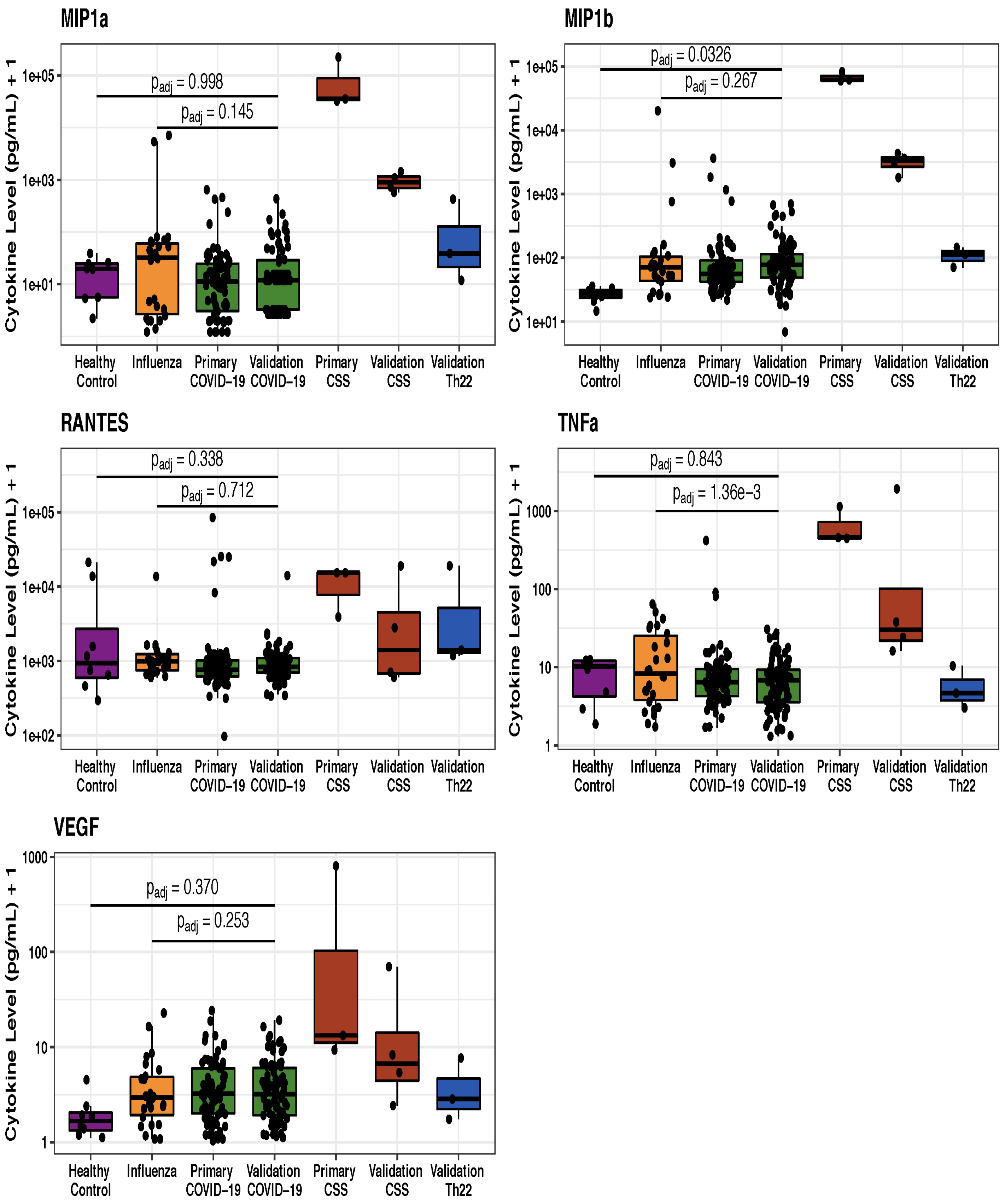

Supplementary Figure 6. Box plots show cytokine concentrations in healthy, influenza, primary COVID-19, validation COVID-19, primary CSS, validation CSS, and validation Th22 groups for all 35 cytokines measured, with raw values plotted on the log10 scale. Presented p-values are from estimated marginal means (EMM) comparisons, averaging over all demographic and clinical factors that were included as covariates and p-values adjusted for multiple comparisons. Although Th22 samples were visualized separately, they were included as validation COVID-19 samples in the underlying statistical analyses.

Sup Fig 7-EMM plots of Flu vs Validation

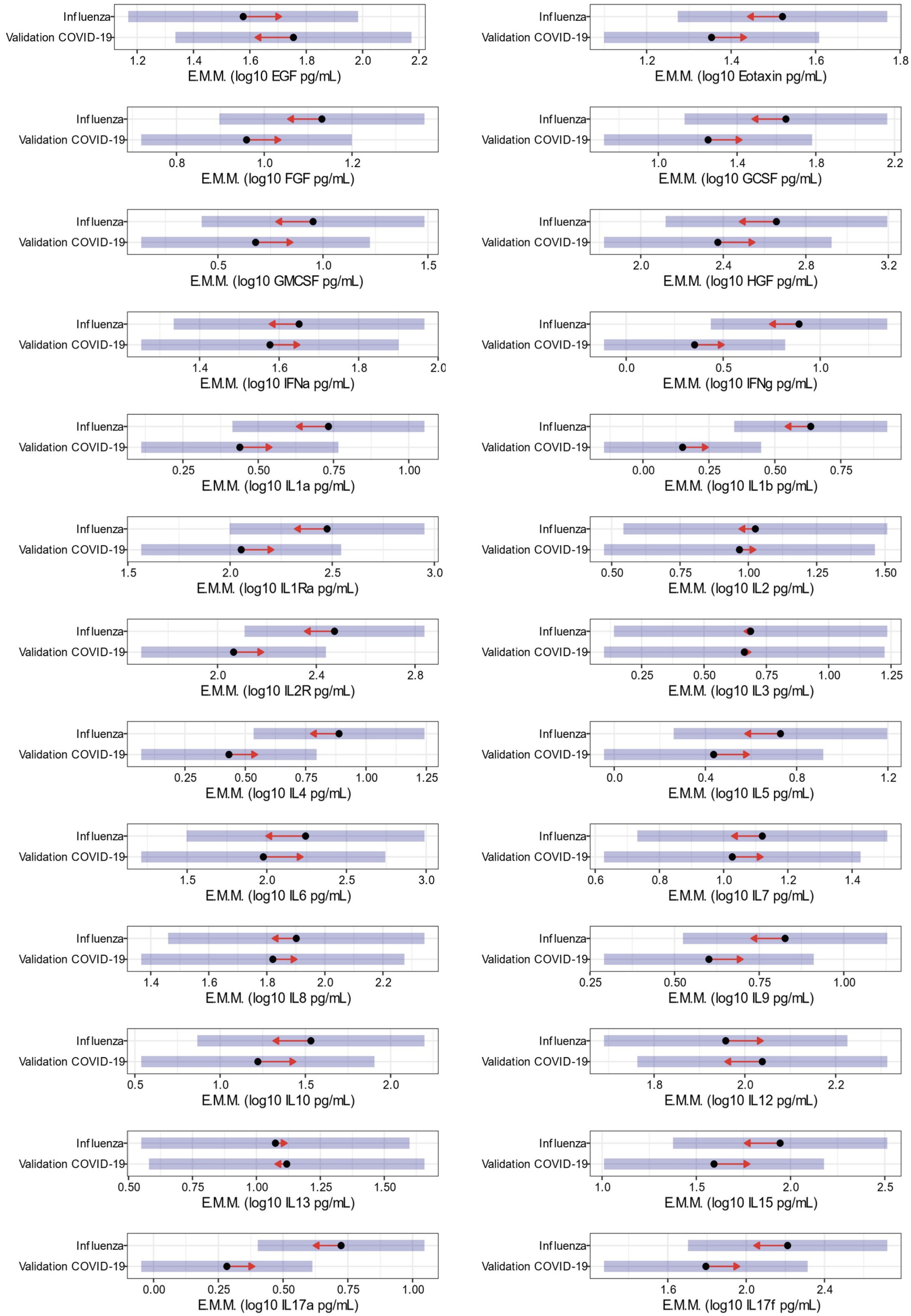

Sup Fig 7 part 2

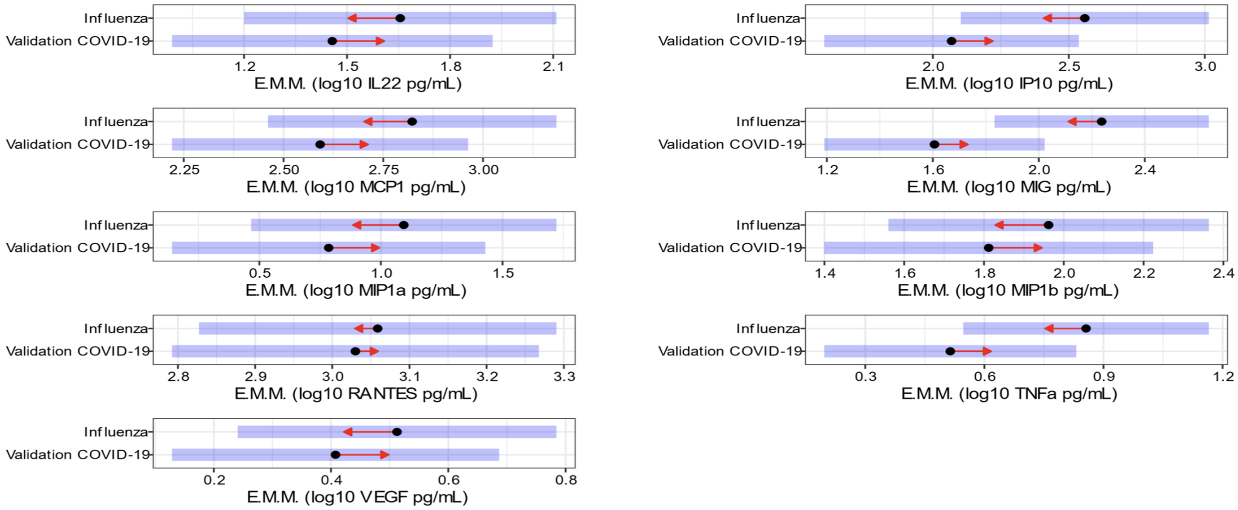

Supplementary Figure 7. Estimated marginal means (EMM) plots comparing cytokine expression between influenza and validation COVID-19 patients. The black dot represents the estimated marginal mean for the log10 concentration of the cytokine for a given condition, averaged over the levels of all other covariates (e.g. age, sex, ethnicity), and the blue shading represents the corresponding 95% confidence Interval. The red arrows represent the standard error (SE) in one direction. Overlapping SE arrows indicate no significant difference between the EMM of a given cytokine in influenza subjects versus COVID-19 subjects.

Supplemental Figure 8.

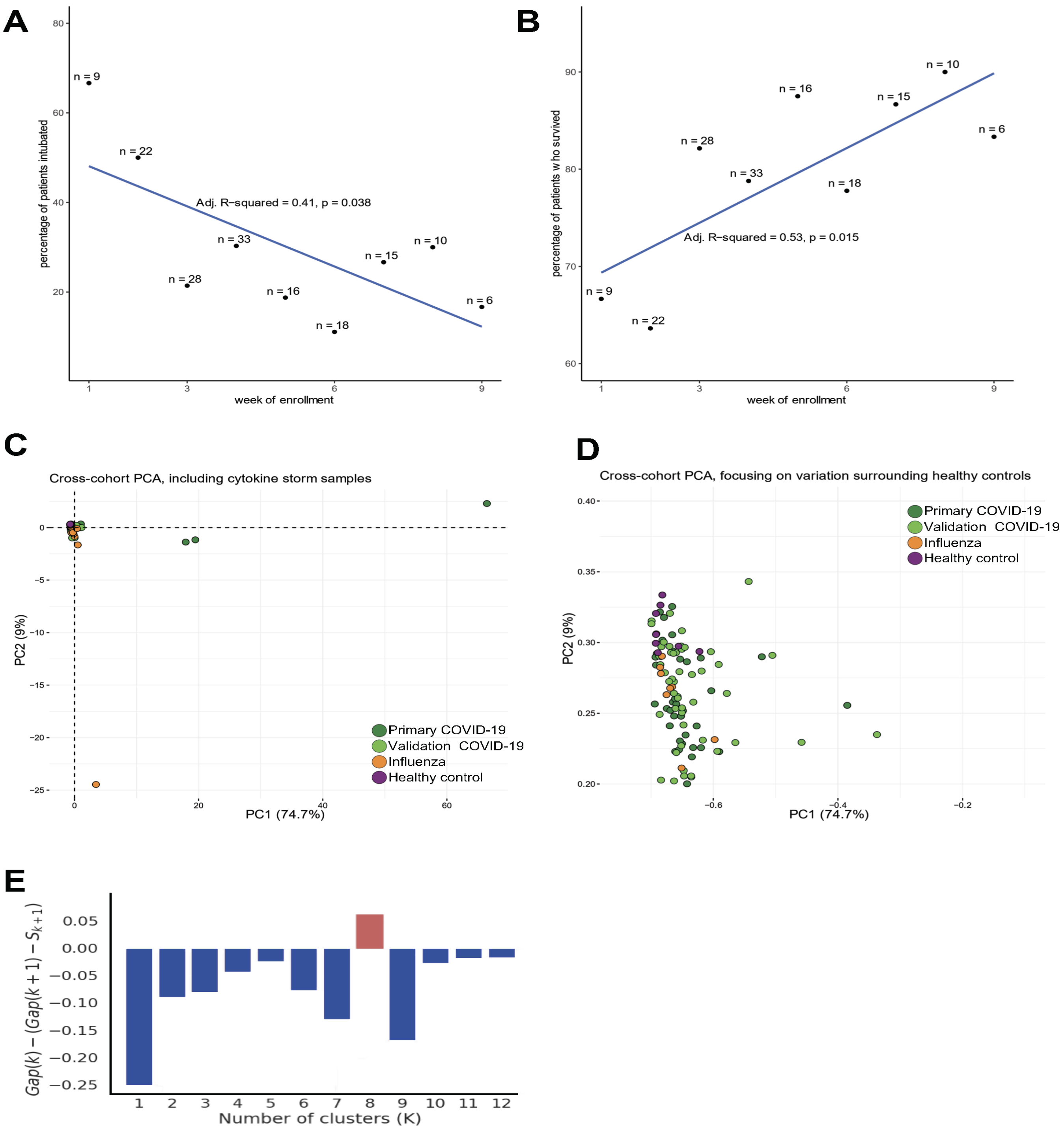

Supplementary Figure 8. Additional cross-cohort comparisons and supporting figures. **(A)** Linear regression of intubation rates binned by week of enrollment shows a significant negative correlation between intubation and time. **(B)** Linear regression of survival rates binned by week of enrollment shows a significant positive correlation between survival and time. **(C)** Principal component analysis (PCA) of all 35 cytokines measured across all patient groups in both the primary and validation cohorts, visualizing all samples with complete cytokine data. **(D)** The same PCA shown in (C) but focused on the variation immediately surrounding healthy controls (i.e., variation owing more extreme values are beyond the bounds of the plot). Magenta circles represent samples from healthy controls, orange circles represent samples from influenza-infected patients, dark green circles represent COVID-19-infected patients from the primary cohort, and light green circles represent COVID-19-infected patients from the validation cohort. **(E)** Change in gap statistic across numbers of clusters (k) for CytoMod module analysis demonstrates an optimal value of k=8.

Supplementary Figure 9

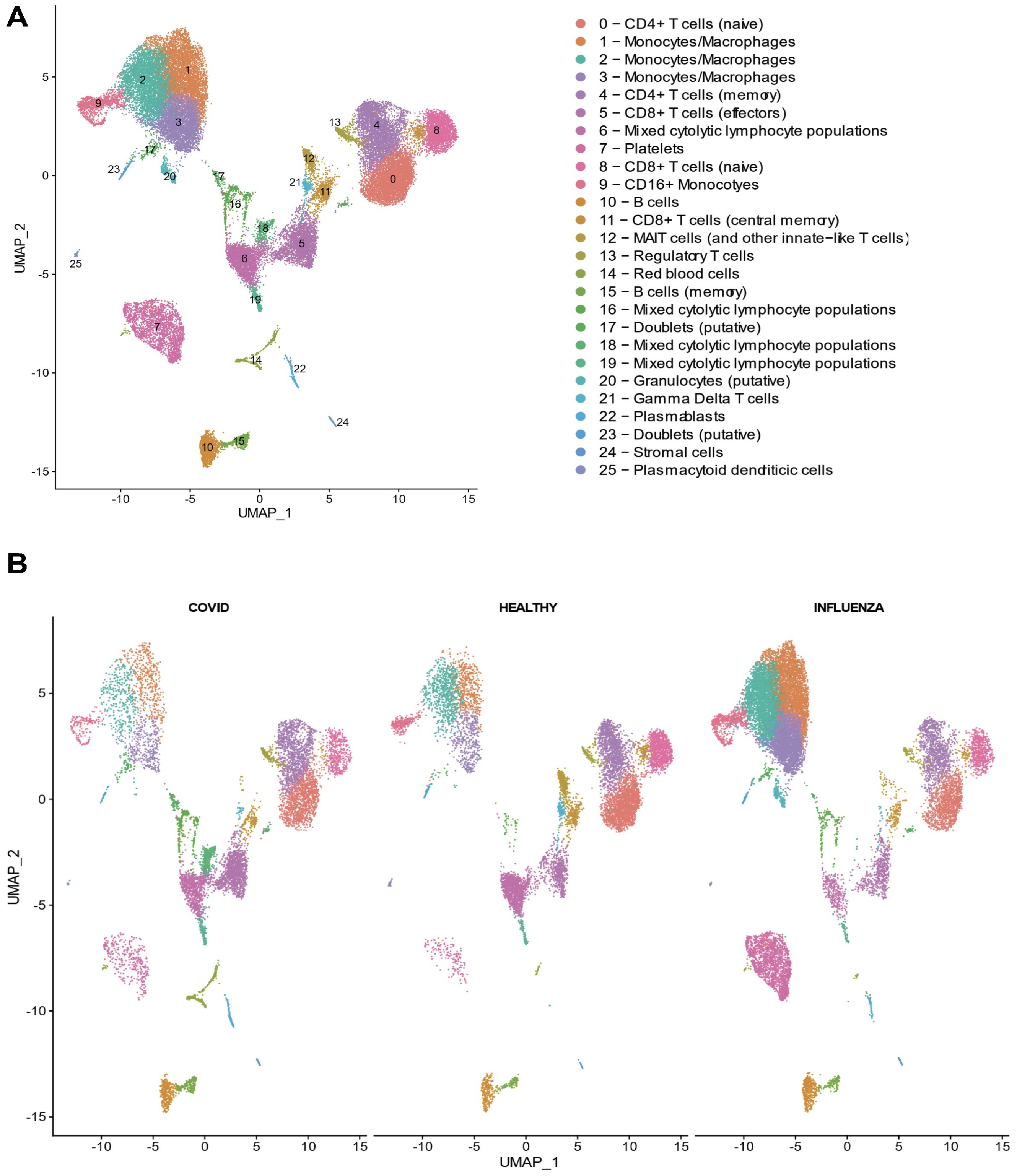

Supplementary Figure 9. Single-cell gene expression analyses of PBMCs from COVID-19-infected, influenza-infected, and healthy subjects demonstrate consistency in major cell subsets despite underlying transcriptional differences across conditions. **(A)** UMAP (uniform Manifold Approximation and Projection) plots depict transcriptional clusters used to define cell subsets. In this case, any differences between subjects has been obscured by merging the data sets with integration anchors via canonical correlation analysis. **(B)** UMAPs split by condition demonstrate the consistency transcriptional cluster identity and presence across conditions.

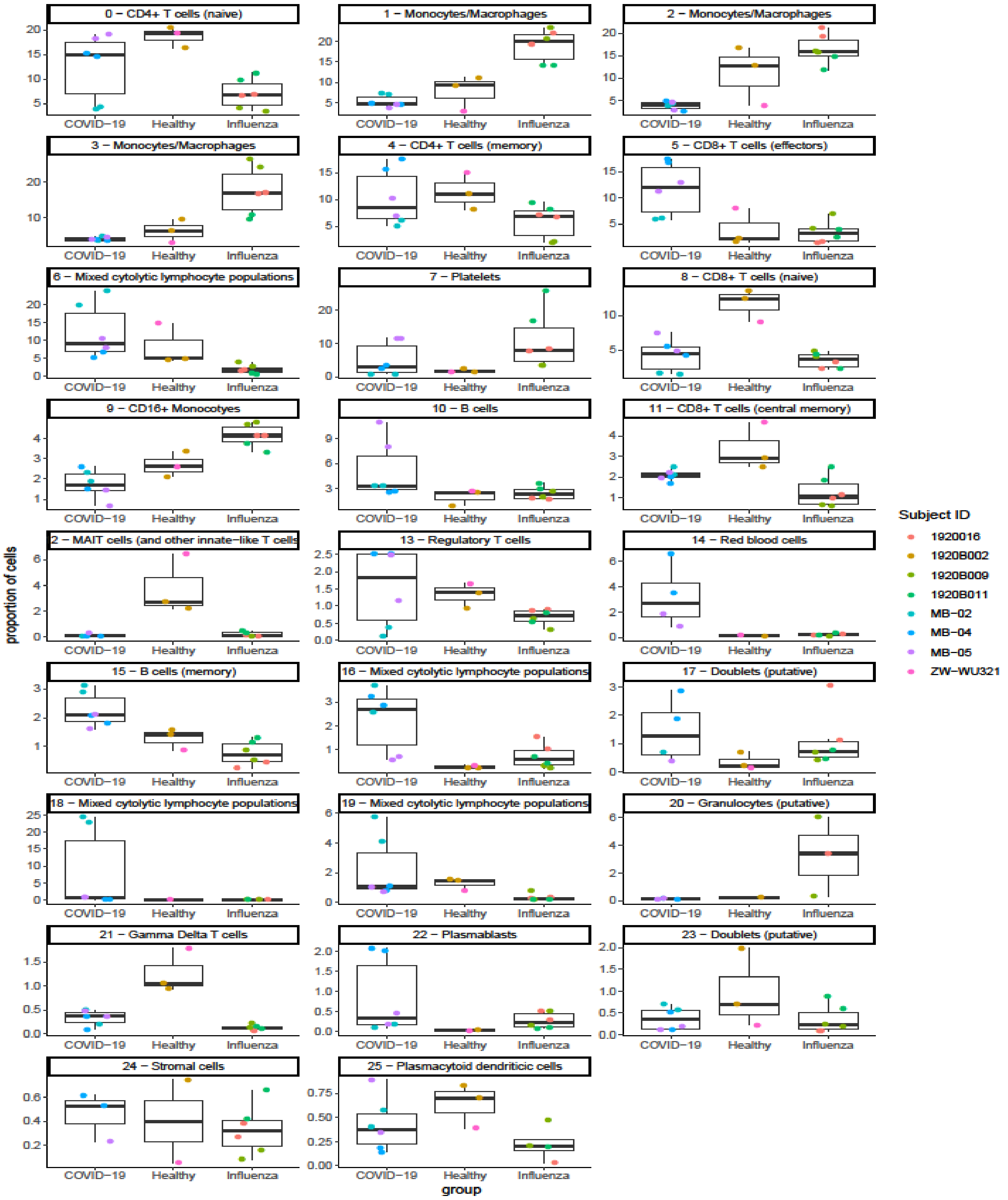

Supplementary Figure 10. For each transcriptionally identified cell subset, the proportion of cells from each single-cell gene expression library that were assigned to that subset is presented. Colors correspond to the patient from which the sample was obtained, with each patient except ZW-WU321 represented by two independent gene expression libraries constructed from distinct cell aliquots from the same PBMC sample (i.e., colors indicate the linked nature of the underlying biological samples). Omission of a point for a cluster indicates failure to detect that cluster in that library.

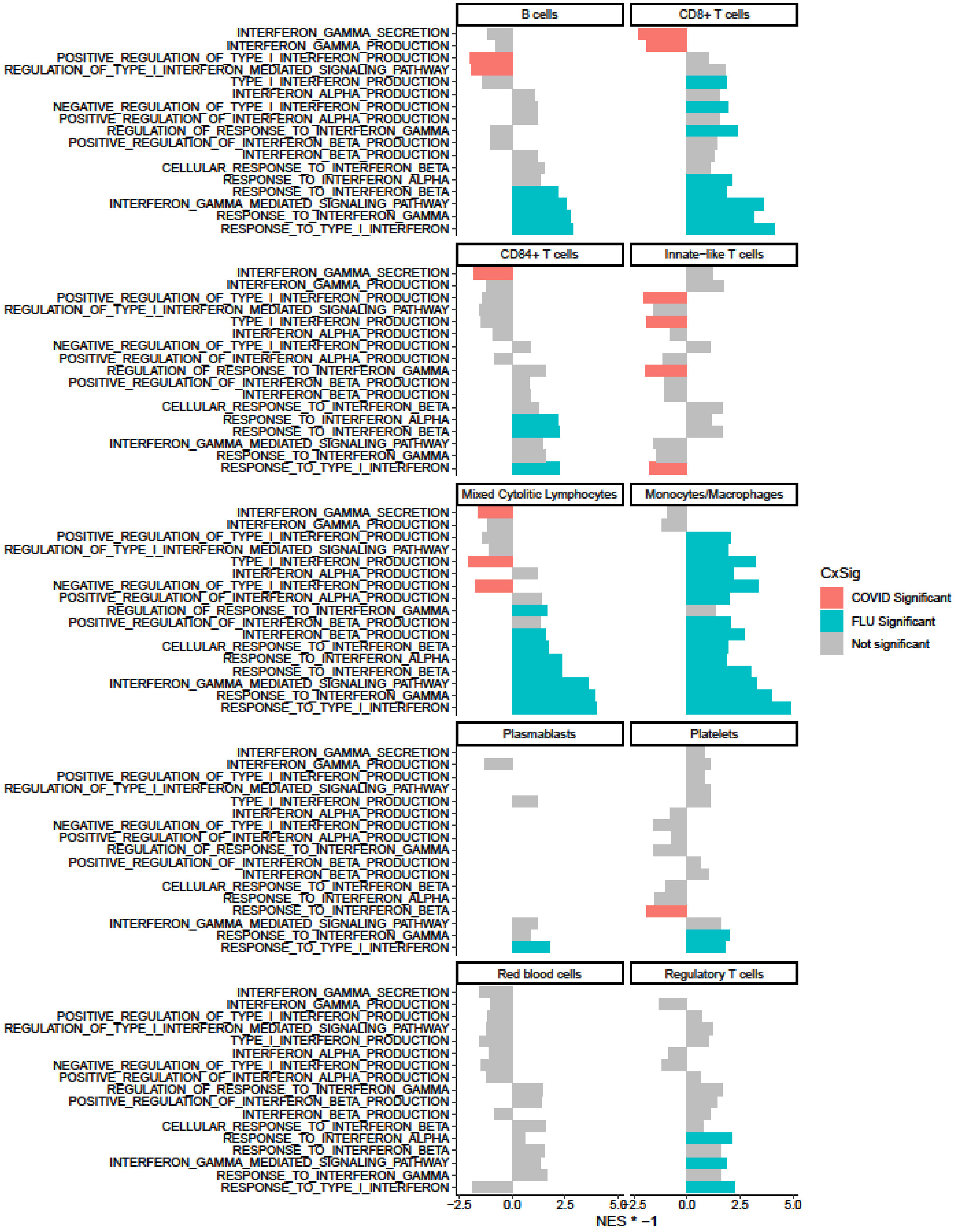

Supplementary Figure 11. Graphs depicting selected results from Preranked Gene Set Enrichment Analysis of Gene Ontology gene sets. Gene Ontology Biological Processes gene sets that passed default GSEA thresholds were tested, with all results with the term “interferon” in the gene set name that were significant in either direction for at least one cell subset plotted. Red: significantly enriched in cells from COVID-19-infected patients; blue: significantly enriched in cells from influenza-infected patients; grey: not statistically enriched in either condition.

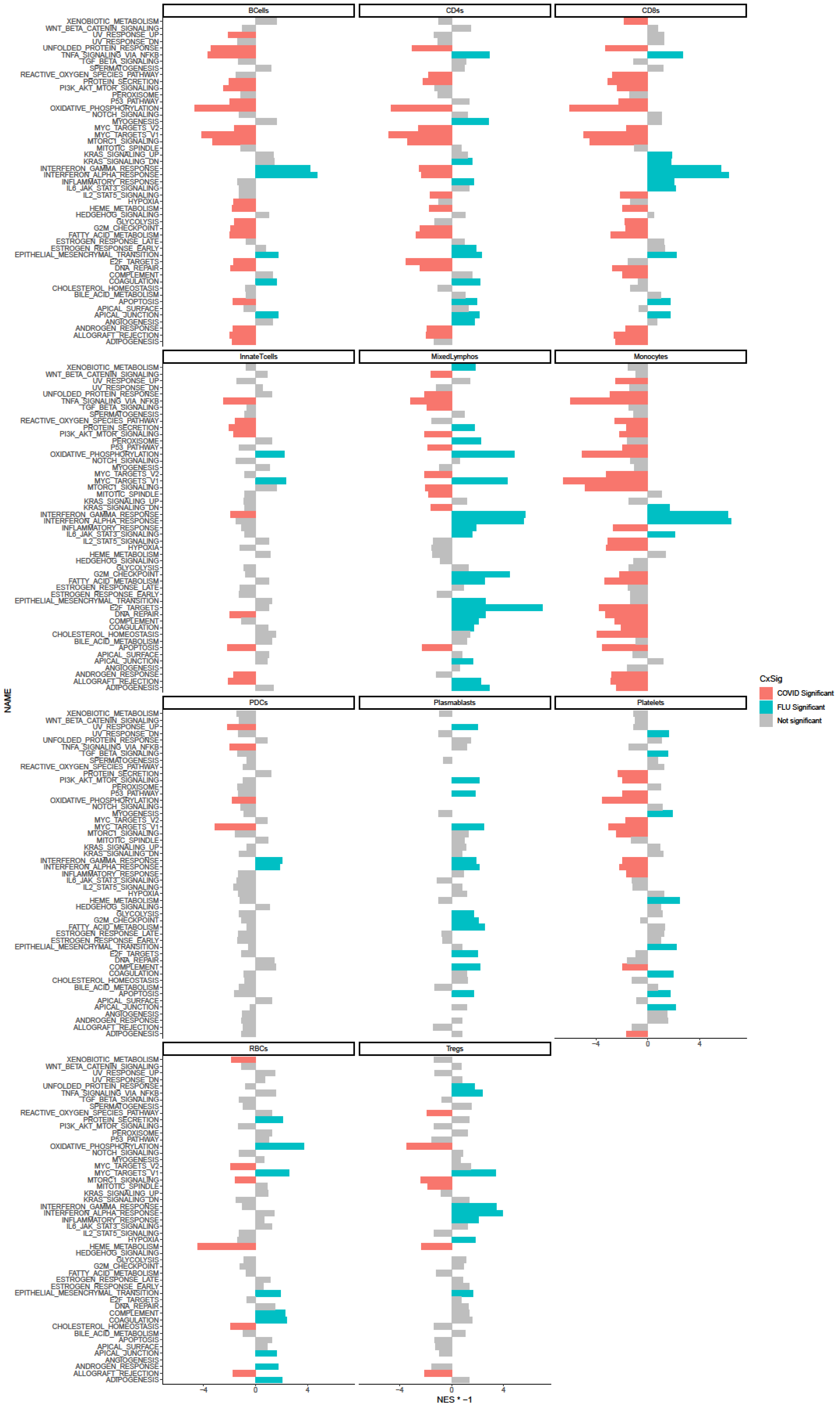

Supplementary Figure 12. Graphs depicting all results of Preranked Gene Set Enrichment Analysis of Hallmark gene sets when comparing COVID-19-infected and influenza-infected subjects on a per-cell-subset basis. Red: significantly enriched in cells from COVID-19-infected patients; blue: significantly enriched in cells from influenza-infected patients; grey: not statistically enriched in either condition.

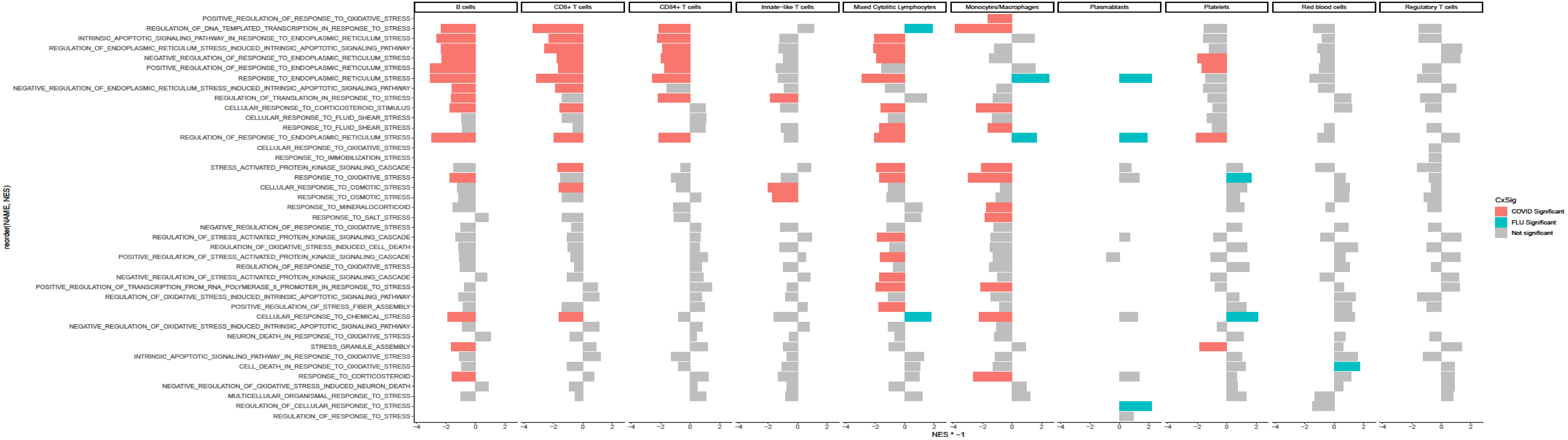

Supplementary Figure 13. Graphs depicting selected results from Preranked Gene Set Enrichment Analysis of Gene Ontology gene sets. Gene Ontology Biological Processes gene sets that passed default GSEA thresholds were tested, and all results with the terms “cortico”, “cortisol”, or “stress” in the gene set name that were significant in either direction for at least one cell subset plotted. Red: significantly enriched in cells from COVID-19-infected patients; blue: significantly enriched in cells from influenza-infected patients; grey: not statistically enriched in either condition.
